# Development and preclinical evaluation of bioactive nerve conduits for peripheral nerve regeneration: A comparative study

**DOI:** 10.1016/j.mtbio.2023.100761

**Published:** 2023-08-05

**Authors:** Elena Stocco, Silvia Barbon, Diego Faccio, Lucia Petrelli, Damiana Incendi, Annj Zamuner, Enrico De Rose, Marta Confalonieri, Francesco Tolomei, Silvia Todros, Cesare Tiengo, Veronica Macchi, Monica Dettin, Raffaele De Caro, Andrea Porzionato

**Affiliations:** a Department of Neurosciences, Section of Human Anatomy, University of Padova, Via Aristide Gabelli 65, 35127, Padova, Italy; b Department of Cardiac, Thoracic and Vascular Science and Public Health, University of Padova, Via Nicolò Giustiniani 2, 35128, Padova, Italy; c L.i.f.e.L.a.b. Program, Consorzio per la Ricerca Sanitaria (CORIS), Veneto Region, Via Nicolò Giustiniani 2, 35128, Padova, Italy; d Plastic and Reconstructive Surgery Unit, University of Padova, Via Nicolò Giustiniani 2, 35128, Padova, Italy; e Department of Civil, Environmental and Architectural Engineering University of Padova, Via Francesco Marzolo 9, 35131, Padova, Italy; f Department of Industrial Engineering University of Padova, Via Gradenigo 6/a, 35131, Padova, Italy

**Keywords:** Peripheral nerve injuries, Nerve conduits, Bioactivation, Nerve regeneration, Self-assembling peptides

## Abstract

In severe peripheral nerve injuries, nerve conduits (NCs) are good alternatives to autografts/allografts; however, the results the available devices guarantee for are still not fully satisfactory. Herein, differently bioactivated NCs based on the new polymer oxidized polyvinyl alcohol (OxPVA) are compared in a rat model of sciatic nerve neurotmesis (gap: 5 mm; end point: 6 weeks). Thirty Sprague Dawley rats are randomized to 6 groups: Reverse Autograft (RA); Reaxon®; OxPVA; OxPVA + EAK (self-assembling peptide, mechanical incorporation); OxPVA + EAK-YIGSR (mechanical incorporation); OxPVA + Nerve Growth Factor (NGF) (adsorption). Preliminarily, all OxPVA-based devices are comparable with Reaxon® in Sciatic Functional Index score and gait analysis; moreover, all conduits sustain nerve regeneration (S100, β-tubulin) without showing substantial inflammation (CD3, F4/80) evidences. Following morphometric analyses, OxPVA confirms its potential in PNI repair (comparable with Reaxon®) whereas OxPVA + EAK-YIGSR stands out for its myelinated axons total number and density, revealing promising in injury recovery and for future application in clinical practice.

## Graphical abstract

**Figure:**
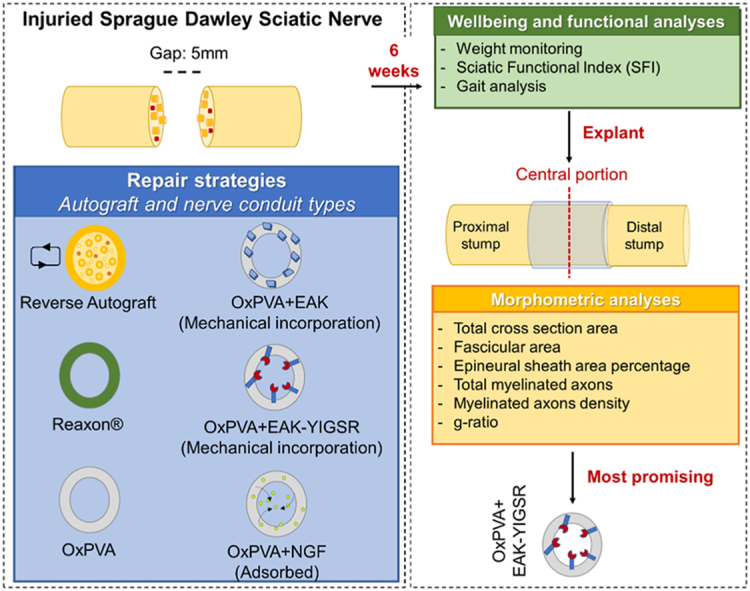

## 1 Introduction

Successful management of peripheral nerve injuries (PNI) is an intriguing challenge in both surgery and research science. Clinical approach strictly depends on injury severity [1]. In case of sharp lesions without substance loss, direct repair (i.e., neurorrhaphy) is commonly performed, also supported by supplementary procedures including protection of the nerve with natural/synthetic wraps to reduce inflammatory/immunologic reaction at the suture site [2]. Differently, in damages with wide interruption of nerve continuity (gap: ≥3 mm), bridging the proximal and the distal stumps is required. To date, to achieve this purpose, the “gold standard” treatment is autograft implantation (mainly sensory nerves like the sural nerve or antebrachial cutaneous nerve); however, drawbacks associated to donor site morbidity, paucity of donor tissue, eventual mismatch between the donor/recipient nerve and the risks/costs associated to second surgery have prompted towards identification of possible alternatives [[3], [4], [5]]. Allografts from cadaver donors can be used; however, despite avoiding the autograft-related issues, they could face host-rejection. Immunosuppression is required up to 18 months post-implantation, in turn increasing patients’ susceptibility towards opportunistic infections that, in the poorer prognosis, may also lead to tumor formation [[6], [7], [8]]. To reduce allograft immunogenicity, decellularization has been adopted (Avance®, AxoGen Inc., Alachua, FL; human origin) but the removal of Schwann cells has limited the use of these products to shorter gaps (up to 3 cm) [9,10].

To overcome the shortcomings related to autografts and allografts, different nerve conduits (NCs) have been developed to provide for safe, ready to use, alternatives. Currently, the Food and Drug Administration (FDA) has approved 11 conduits for repair of PNI. These are made of biomaterials of both natural (collagen type I, extracellular matrix from porcine small intestine submucosa, chitosan) and synthetic (polyvinyl alcohol (PVA), polyglycolic acid (PGA), poly(d,l-lactide-co-ε-caprolactone) (PLCL)) origin that, except for the PVA-based one (SaluTunnel, non-biodegradable), show a certain degradation profile ranging from 3 months (NeuroTube, PGA) to 48 months (NeuraWrap, Neuragen, collagen type I) and likely consistent with nerve regeneration time [[11], [12], [13]]. Even though NCs' advantages are a significant decrease in time of surgery and no further impact on patients’ health, the results they guarantee for are still not fully satisfactory. Evidence shows that they may fail to regenerate and recover the complete nerve function with performances inferior to that of autografts.

Mechanical rigidity, poor fit to the end-stump, lacking customization are among the weaknesses of the current devices, thus boosting research into material sciences, biotechnologies and tissue engineering towards the identification of effective alternatives. Specifically, ideal substitutes are expected not only to behave as a protective physical space but also undertake multiple functions to speed up nerve regeneration [14]. The translational design criteria they are expected to show include: i) sufficient availability; ii) tailored size/diameter; iii) mechanical strength; iv) biodegradability; v) no toxicity/immunogenicity; vi) wall permeability; vii) bioactivity [15].

Oxidized polyvinyl alcohol (OxPVA) is a synthetic polymer developed by our group; experimental evidences broadly showed its promising characteristics for tissue engineering purposes [[16], [17], [18]], including fabrication of devices supporting peripheral nerve regeneration (nerve conduits and wraps) [2,[19], [20], [21], [22]]. By virtue of OxPVA chemistry, the derived scaffolds can act as customizable platforms: introduction of 1% carbonyl groups in the polymer backbone not only guarantees for a certain biodegradation rate after crosslinking [17], but also allows for protein loading by polymer/proteins Schiff-base interactions [17,19,21] or covalent binding [17]. Additionally, OxPVA biochemical activation by mechanical incorporation of peptide cues (Self Assembling Peptide (SAP) EAK) has been recently exploited too, with encouraging in vitro results on neuronal SH-SY5Y cells proliferation [23].

The SAPs are a new class of compounds that can spontaneously give rise to stable α-helix, β-sheet, or random coil, in turn organizing into 3D aggregation states resembling extracellular matrix (ECM) ultrastructure. Due to this specific behaviour, they revealed important in the field of nanotechnology and biotechnology for possible application in tissue engineering [24,25]. In particular, ionic-complementary peptides, characterized by a sequence that sees the alternation of hydrophobic and charged amino acids, have found use as scaffolds for the growth of bone, cartilage and nervous tissue cells [26]. Furthermore, the possibility of obtaining these sequences by synthesis offers the opportunity to add signal sequences for the adhesion and growth of specific cell populations or functional groups for specific and selective anchoring to other materials or surfaces. Among functional specific motifs holding great clinical promise for PNI repair we can cite IKVAV: isoleucine–lysine–valine–alanine–valine; RGD: arginine-glycine-aspartic acid; YIGSR: tyrosine–isoleucine–glycine–serine–arginine [27,28].

Considering SAPs and SAPs-conjugates potential, together with consciousness on PNI challenging impact on health, the goal of this study was to enhance the neuro-conductive microenvironment of OxPVA-based NCs: bioactive molecules (EAK or its laminin-derived conjugated EAK-YIGSR) or growth factors (Nerve Growth Factor (NGF)) were mechanically incorporated or adsorbed, respectively, within the devices. Thus, the enriched NCs were compared to others including OxPVA alone, Reverse Autograft (RA) and Reaxon® (based on chitosan; Keri Medical, Switzerland) in an animal model of severe PNI (gap: 5 mm). The repair efficiency of OxPVA-based hollow NCs was confirmed at 6 weeks from surgery; the study results may lay the basis for future use of OxPVA-derived devices in clinical treatment of PNI.

## 2 Experimental section

### 2.1 Materials

The solid support resin Rink Amide MBHA, the Fmoc protected amino acids, the coupling reagents 2-(1H - Benzotriazole-1-yl) −1,1,3,3-tetramethyluronium hexafluorophosphate (HBTU), ethyl cyano(hydroxyimino)acetate (oxyma pure), *N*,*N*-diisopropylethylamine (DIEA), triethylsilane (TES), *N*,*N*-dimethylformamide (DMF) and piperidine were from 10.13039/100009945Merck KGaA, (Darmstadt, Germany). Trifluoroacetic acid (TFA), diethyl ether and dichloromethane (DCM) were from Biosolve (Leenderweg, Valkenswaard, Netherlands). Acetonitrile was from Carlo Erba (Milan, Italy). *N*-methyl-2-pyrrolidone (NMP) was purchased by Iris Biotech GmbH (Halle, Germany).

### 2.2 Synthesis of the self-assembling peptide (SAP) EAK and its rhodamine-conjugated analog (EAK-Rh)

The synthesis, purification and characterization of EAK and EAK-Rh were performed as previously reported [23]. Briefly, EAK (sequence: H-Ala-Glu-Ala-Glu-Ala-Lys-Ala-Lys-Ala-Glu-Ala-Glu-Ala-Lys-Ala-Lys-NH_2_) was synthesized on Rink Amide MBHA resin (0.52 mmol/g) using Fmoc chemistry by a Syro I synthesizer (Multisyntech, Witten, Germany). The side-chain protecting groups were: OtBu, Glu and Boc, Lys. All the couplings were double (for each coupling 5 equivalents of Fmoc-amino acid, 5 eq. HBTU, 5 eq. oxyma pure and 10 eq. DIEA were used). After Fmoc-deprotection of the last inserted amino acid, the peptide was deblocked from the resin and deprotected from side-chain protecting groups using the mixture 1.9 mL TFA, 0.05 mL TES, 0.05 mL H_2_O, for 1.5 h. The resin was filtered off and the solution was concentrated and added with cold diethyl ether. The product was precipitated and filtered. The identity of the crude peptide was determined by mass spectrometry (exp. mass = 1614.52 Da; theor. mass = 1614.79 Da; AB-SCIEX TOF-TOF 4800 instrument). The peptide EAK was isolated by RP-HPLC and characterized by analytical RP-HPLC (conditions: Vydac C_18_ column (5 μm, 300 Å, 4.6 × 250 mm, Grace), eluent A: 0.05% TFA in H_2_O; eluent B: 0.05% TFA in CH_3_CN; gradient: from 5 to 20% B in 30 min, flow rate: 1 mL/min; detector: 214 nm. t_R_ = 21.62 min). The integration of the chromatogram confirmed a 94% purity grade.

The EAK-Rh preparation consisted of the reaction between side chain-protected EAK peptide on resin and 4 equivalents of 5(6)-carboxy tetramethylrhodamine (Merck KGaA), 4 equivalents of HBTU and 8 equivalents of DIPEA in DMF for 45 min. The crude peptide was deprotected and cleaved from the resin as above reported and isolated by RP-HPLC.

### 2.3 Synthesis of the SAP EAK-YIGSR

The synthesis, purification and characterization of EAK-YIGSR were performed as previously reported [29]. Briefly, the peptide EAK-YIGSR (sequence: H-Ala-Glu-Ala-Glu-Ala-Lys-Ala-Lys-Ala-Glu-Ala-Glu-Ala-Lys-Ala-Lys-Tyr-Ile-Gly-Ser-Arg-NH_2_) was synthesized on Rink Amide MBHA resin (0.52 mmol/g) using Fmoc chemistry by a Syro I synthesizer (Multisyntech, Witten, Germany). The side-chain protecting groups were: OtBu, Glu; Boc, Lys; Pbf, Arg; *t*Bu, Ser and Tyr. All the couplings were double. After Fmoc-deprotection, the peptide was deblocked from the resin and deprotected from side-chain protecting groups using the mixture 1,9 mL TFA, 0.05 mL TES, 0.05 mL H_2_O, for 1.5 h. The resin was filtered off and the solution was concentrated. The product was precipitated with diethyl ether and filtered. The identity of the crude peptide was determined by MALDI mass spectrometry (exp. mass = 2192.06 Da; theor. mass = 2191.45 Da; AB-SCIEX TOF-TOF 4800 instrument). The peptide EAK-YIGSR was purified by RP-HPLC and characterized by analytical RP-HPLC (conditions: Nova-Pak HR C_18_ column (4 μm, 60 Å, 3.9 × 300 mm, Waters), eluent A: 0.05% TFA in H_2_O; eluent B: 0.05% TFA in CH_3_CN; gradient: from 18 to 26% di B in 24 min, flow rate: 1 mL/min; detector: 214 nm. t_R_ = 10.68 min). The integration of the chromatogram gave a 99% purity grade.

### 2.4 Preparation of oxidized polyvinyl alcohol solution

The solution of OxPVA was prepared in accordance with Stocco et al. [17]. Briefly, 10 g of PVA powder [molecular weight (Mw) 146 000–186 000 Da, 99+% hydrolysed] were suspended in MilliQ water; solubilization was achieved by heating the system in boiling bath under stirring for 1 h. Hence, the solution was cooled at 37 °C and polymer partial oxidation took place through the addition of an acidic potassium permanganate (KMnO_4_) solution (151 mg of KMnO_4_ in 10 mL of MilliQ water + 1.60 g of 70% perchloric acid (HClO_4_) (w/w)). Oxidative reaction run for 1 h at 37 °C; thereafter, extensive dialysis was performed (8000 Da cut-off (Sigma-Aldrich)). OxPVA polymer solution was then frozen at −20 °C overnight and lyophilized (Speed Vac Concentrator Savant, Instruments Inc., Farmingdale, NJ, USA) for long-term storage. For polymer recovery, 16 wt (wt) % OxPVA was weighted, suspended into MilliQ water, and heated in boiling bath.

### 2.5 OxPVA-based nerve conduits fabrication

Four different types of OxPVA-based nerve conduits were here developed: a) OxPVA; b) OxPVA + EAK; c) OxPVA + EAK-YIGSR; d) OxPVA + NGF.

The SAPs EAK and EAK-YIGSR were mechanically incorporated within the polymer solution, prior to proceed with nerve conduits fabrication; conversely, the NGF was used as pre-made OxPVA conduits conditioning agent, by adsorption.

#### 2.5.1 OxPVA NCs

A 16% (wt/wt) OxPVA polymer solution was reconstituted in MilliQ water as previously described and it was poured into a tubular stainless mold (internal diameter: 2.1 mm) equipped with a central coaxial mandrel (diameter: 1.2 mm); thereafter, polymer crosslinking was achieved by freezing (at −20 °C for 24 h)/thawing (at 2.5 °C for 24 h) (FT): seven FT cycles were required for NCs fabrication. The NCs were stored at 0 °C until use.

#### 2.5.2 OxPVA + EAK and OxPVA + EAK-YIGSR NCs

The EAK and EAK-YIGSR powders were resuspended in MilliQ water (1 mg/mL); in parallel, 3 g of OxPVA hydrogel were poured onto a glass slide and EAK or EAK-YIGSR were added drop by drop (0.2% wt/wt), respectively. Homogeneous distribution of the peptides solution within the hydrogel was assured by mechanical incorporation using a stainless laboratory spatula. Thereafter, the SAP-enriched hydrogels were cast into the moulds as previously described and exposed to FT. The NCs were stored at 0 °C until use.

#### 2.5.3 NGF-conditioned NCs

OxPVA + NGF NCs were prepared through adsorption; OxPVA conduits, set-up as previously described, were incubated for 24 h at 37 °C in 0.5 mL of phosphate buffer (PBS) solution containing 0.1% (wt/wt) BSA and 125 μg/mL NGF. Conditioning was performed immediately before the implant.

Prior to use, the OxPVA-based devices were decontaminated with 1% (wt/wt) antibiotic solution and carefully washed with PBS (0.1 M, pH 7.4).

### 2.6 OxPVA-based conduits ultrastructural characterization

OxPVA nerve conduits were investigated for their ultrastructure; in particular, the lumen and the conduit wall were focused. The samples were fixed with 2.5% glutaraldehyde solution in 0.2 M PBS (pH 7.4) for 24 h, washed 5 times in PBS to remove chemical residues and then dehydrated with a graded ethanol series. Following critical point drying and gold sputtering, micrograph acquisition was performed by using the tungsten thermionic emission SEM system JSM-6490 (Jeol USA, Peabody, MA, USA).

### 2.7 Validation of peptide mechanical incorporation

The EAK-Rh powder was resuspended in MilliQ water to a final concentration of 1 mg/mL; in parallel, 3 g of OxPVA hydrogel, pre-heated at 45 °C, were poured onto a glass slide. Hence, the EAK-Rh solution (0.2% wt/wt) was added drop by drop to the polymer and incorporated homogeneusly using a stainless laboratory spatula. Thereafter, the OxPVA + EAK-Rh solution was poured into a tubular mold for nerve conduits fabrication and the system underwent to FT as previously described. The derived scaffolds were observed through a custom-developed multiphoton microscope [30]. Non-functionalized OxPVA conduits were used as negative controls.

Quantification of EAK-Rh peptide embedded into OxPVA (C_eq_) was performed using a method already reported [31,32] and was based on two-photon microscopy acquired images. The calibration curve was obtained with serial dilutions of free EAK-Rh in PBS (from 6 × 10^−6^ M to 6 × 10^−9^ M). The intensity, in the red channel, was measured through Fiji software [33] of three acquired images for each dilution of the standard curve. Average values with the corresponding standard errors were used to build the calibration curve and PBS was set as blank. Linear fit were applied and the extrapolated equation was employed to calculate the concentration of the EAK-Rh peptide embedded into the scaffolds.

### 2.8 Evaluation of NGF kinetic release profile

NGF kinetic release profile was determined considering 2D-samples (diameter: 7 mm, thickness: 0.6 mm) preliminarily obtained using a biopsy punch from an OxPVA polymer membrane, prepared through FT (see protocol in 2.5.1.). The mold used for membrane fabrication (i.e., two steel sheets: 10 cm in height x 10 cm in width; thickness of each sheet: 0.44 mm; spacer thickness: 0.6 mm) allowed to guarantee for scaffolds showing the same characteristics of the conduits.

NGF was adsorbed to OxPVA 2D-samples at 0.125 mg/mL (300 μL) concentration, following the protocol described in 2.5.3 paragraph. Following scaffolds conditioning, each sample (triplicate) was then incubated with 200 μL of PBS. The solution was picked out at 10 min, 20 min, 30 min, 1 h, 2 h, 6 h, 24 h and substituted with fresh PBS (200 μL). Thereafter, to determine NGF kinetic release profile, reversed phase high-performance liquid chromatography (RP-HPLC) was adopted. All chromatographic runs were carried out in the same conditions: column Symmetry Shield RP8 C8 (5 μm, 100 Å, 4.6 × 250 mm, Waters); Eluent A, 0.05% TFA in MilliQ water; Eluent B, 0.05% TFA in acetonitrile; gradient from 15% to 55% of B in 40 min; flow rate, 1 mL/min; detection at 214 nm (HPLC Waters 1525, 717 Autosampler, 996 UV/Vis detector, Waters Corporation, Milford, Massachusetts, USA).

A calibration curve was built to then determine the released NGF levels from OxPVA scafffolds.

### 2.9 OxPVA-based conduits mechanical testing

Uniaxial tensile tests were carried out on rectangular samples with 1:3 width-to-free length ratio (width 8 mm × free length 24 mm) and a thickness of 0.6 mm, corresponding to the wall thickness of the nerve conduits (the mold used, from which the samples were excised, was the same described in paragraph 2.8). Eight samples for each material (OxPVA; OxPVA + EAK; OxPVA + EAK-YIGSR; OxPVA + NGF) were tested.

Tests were run at a strain rate of 0.5%s^−1^, up to 100% of strain, on Bose ElectroForce® Planar Biaxial Test Bench (TA Instruments, New Castle, USA), with a load cell of 22 N. The hydration of the samples was ensured during the test by dropping PBS solution on the sample surface. Tests were carried out with displacement control, acquiring the corresponding force data. The nominal stress σ was calculated as the ratio between current force and initial transversal area of the sample (initial width × thickness), while nominal strain was calculated dividing the imposed elongation by the initial length of the sample. For each experimental group mean, values of nominal strain and standard deviations (SD) were calculated. Secant elastic modulus was obtained from the nominal stress versus strain data of each sample, at a strain level of 20%.

### 2.10 Microsurgical technique

Animal surgery and husbandry were performed in accordance with the Italian guidelines on the use of experimental animals and approved by the Ethical Committee of the University of Padua and by the Italian Department of Health (Authorization n. 837/2019-PR, December 09, 2019).

Thirty Sprague-Dawley rats (male) were randomly divided into six experimental groups (n = 5/each): RA (positive control), Reaxon® (commercial product based on chitosan), OxPVA, OxPVA + EAK, OxPVA + EAK-YIGSR and OxPVA + NGF groups. After anesthesia administration, consisting in a gas mixture of isoflurane/oxygen, the left thight was shaved and disinfected. Hence, the sciatic nerve was exposed through a gluteal-splitting approach and transected leaving a gap of 5 mm between the proximal and distal nerve stumps. Regarding the RA group, a segment of sciatic nerve (5 mm in length) was excised, inverted and reimplanted using Nylon 8–0 sutures. As for the conduits (10 mm in length), these were coaxially interposed between the stumps and sutured to the epineurium using Nylon 8–0 sutures. The incision was closed in layers through 4–0 silk sutures. After surgery, the animals recovered in the cage; hence, anti-inflammatory (Rimadyl®, 5 mg/kg) and antibiotic (Baytril®, 5 mg/kg) therapies were administered for 5 days after implant. During the experimental period, rats were housed in a temperature-controlled facility, fed with laboratory rodent diet and given water ad libitum.

After 6 weeks, euthanasia was induced through carbon dioxide asphyxiation; hence, the implants were excised, preliminarily analyzed for their size/integrity and then properly fixed for histological/immunohistochemical analyses and SHG (n = 2/group) as well as for semithin sections preparation and TEM (n = 3/group).

### 2.11 Animals wellbeing assessment and behavioral tests of motor function

#### 2.11.1 Weight analysis

At 1, 3 and 6 weeks after surgery, rats' weight was monitored to verify their wellbeing. As weight is a great indicator of animals’ health, evident variations suggest poor toleration of the surgical procedure.

#### 2.11.2 Sciatic functional index

At 6 weeks after surgery, the functional status of the operated sciatic nerve was assessed evaluating the sciatic functional index (SFI). Briefly, as previously described [2] a gangway lined with white paper was set-up, the rats’ posterior paws were stained with black ink and thus the animals were placed at the beginning of the lane to walk along it. Thus, the SFI was calculated according to Bain et al. [34] considering this formula:

***SFI*** = [− 38.3 x (*EPL*−*NPL*)/*NPL*] + [109.5 x (*ETS*−*NTS*)/*NTS*] + [13.3 x (*EIT*−*NIT*)/*NIT*] − 8.8

Print length (PL) being the distance from the heel to the top of the third toe; toe spread (TS), the distance from the first to the fifth toe; intermediary toe spread (IT), the distance between the second and the fourth toe. NPL, NTS and NIT represent the PL, TS and IT referring to the non-operated foot. EPL, ETS, and EIT represent the PL, TS and IT referring to the operated foot (E, experimental side). Score around 0 corresponds to normal nerve function; score around −100 corresponds to total motor sciatic nerve dysfunction.

#### 2.11.3 Gait analysis

At 6 weeks after surgery, before euthanasia, the animals were walked down a transparent plexiglass lane (50 cm long and 10 cm wide), darkened at one end; below it, it was placed a 45° inclined mirror. This set-up allowed to simultaneously view the lateral profile of the animal and the operated-paw support, during the gait cycle. The walk of each rat was videotaped for groups comparisons. Specifically, the considered parameters included: toe spread; walking on plantar side; absence of dragging; normal swing phase; fluent walking; absence of eversion; absence of exrotation; alternating steps; hindfoot within body perimeter; joint contraction [35]. For each of the above parameters, it was assigned a score as follows: 0, not assessable; 1, assessable but abnormal; 2, normal. The maximum mean score/animal attributable was 2.0; a mean total score was attributed to each animal/group and the groups mean score was then compared.

### 2.12 Characterization studies for neuropathological analyses

Once the animals were euthanized, the implants were evaluated both in site and after their removal through different methodological approaches.

#### 2.12.1 Evaluation of the surgical site

After dissection, the surgeon performed a preliminary blind evaluation of the neurolysis site considering eventual inflammation and perineural adhesion presence. Adherences were classified in accordance with Petersen et al. [36], as reported in Table 1.

**Table 1:** Adherences scoring and description of the severity grade.

Grade of severity	Description
1	Minimal adhesion that did not require dissection or that required minimal dissection
2	Moderate adhesions that required some vigorous blunt dissection but without sharp tools
3	Significant adhesions that required sharp dissection

#### 2.12.2 Histological and immunohistochemical analyses

Once harvested, the samples were fixed in 10% (wt/v) formalin in PBS (n = 2/group); hence, 4 μm-thick serial sections were obtained, after paraffin embedding. From each experimental group, both longitudinal and transversal sections were prepared. Longitudinal sections were stained by H&E according to routine protocols. In parallel, cross-sections referring to the middle samples’ portion underwent immunological characterization through the following antibodies diluted in PBS: anti-CD3 (polyclonal rabbit anti-human CD3, A 0452; Dako®, Milan, Italy) diluted 1:500; anti-F4/80 (polyclonal rabbit anti-mouse anti-F4/80, sc-26643-R; Santa Cruz Biotechnology, CA, USA) diluted 1:800; anti S-100 (polyclonal rabbit anti-S100, Z 0311; Dako®) diluted 1:5000; anti-β-tubulin (polyclonal rabbit neuronal class III β-tubulin, PRB–435 P; Covance, Princeton, NJ, USA). Except for S-100, antigen unmasking was performed (10 mM sodium citrate buffer, pH 6.0, at 90 °C for 10 min); hence, the sections were incubated in blocking serum [0.04% (wt/v) bovine serum albumin (BSA; A2153, Sigma-Aldrich, Milan, Italy) and 0.5% (wt/v) normal goat serum (X0907, Dako®)] for unspecific binding removal (30 min at room temperature, RT) and then with the above primary antibodies (1 h at RT). Primary antibody binding was revealed by incubation with anti-rabbit/mouse serum diluted 1:100 in blocking serum (30 min at RT) (Dako® EnVision + TM peroxidase, rabbit/mouse; Dako®, Glostrup, Denmark) and developed in 3,3-diaminobenzidine (3 min at RT). Haematoxylin was used for counterstaining. As a negative control, incubation without primary antibodies was performed.

### 2.13 Morphometric analysis

Morphometric analysis was performed to assess axonal regeneration degree and thus compare devices efficiency. Briefly, as previously described [20], samples (n = 3/group) were preliminarily fixed in 2.5% (wt/v) in glutaraldehyde in 0.1 M PBS, divided in different segments (from 4 to 5, allowing to clearly identify the centre of the guide) and post-fixed in 1% osmium tetroxide (Agar Scientific Elektron Technology - UK) in 0.1 M phosphate buffer. Hence, segments were dehydrated in a graded alcohol series and embedded in Epoxy resin. Semi-thin sections (0.5 μm) were cut with an ultramicrotome RMC-PTX PowerTome (Boeckeler Instruments, Arizona-USA) from the central segment and stained with 1% toluidine blue. Images were acquired by using Leica DMR microscope (Leica Microsystems Wetzlar- Germany). In parallel, ultrathin sections, 60 nm, were collected on 300-mesh copper grids, counterstained with 2% (wt/v) uranyl acetate and then with Sato's lead. Specimens were observed by a Hitachi H-300 TEM.

Morphometric study was performed on semithin toluidine blue stained sections. The central portion of each sample was considered to measure the mean total cross section area [μm^2^] and fascicular area [μm^2^]; hence, the epineural sheath area (%) was inferred as follows: ([total cross section area – fascicular area] x 100). Subsequentially five random quadrants in the fascicular area were identified and three high power fields (7523 μm^2^/quadrant) were investigated for total myelinated axons number, average myelinated axons density (axons/μm^2^) and g-ratio. The g-ratio (degree of myelination) was calculated by measuring the diameter of axons and dividing by the total diameter of that axon plus the surrounding myelin sheath (fiber diameter). All measurements were performed using ImageJ software. Three animals/group were considered for the morphometric study. For each animal, 15 images were used.

Samples processing for morphometric studies was assigned to a single, expert, research-collaborator to exclude operator-dependent variability.

### 2.14 SHG analysis

Second Harmonic Generation Microscopy, performed through a custom-developed multiphoton microscope [30], was adopted for identification of fibro-connective infiltrate within the samples. As previously described [37], an incident wavelength of 800 nm (∼40 mW average laser power, under the microscope objective) was applied to detect the collagen's SHG signal at 400 nm. Images acquisition was performed at a fixed magnification by an Olympus 25× water immersion objective with 1.05 numerical aperture (1024 × 1024 pixels), averaged over 100 consecutive frames, with a pixel dwell time of 0.14 μs and a pixel width of 0.8 μm. Additionally, for a broader samples' description, organization/distribution of the fibers was also investigated determining the Coherency (C) by FFT [38] and OrientationJ [39]. The C parameter is bounded between 0 and 1, indicating the absence (isotropy) and the presence (anisotropy) of dominant orientation, respectively. Regarding the FFT, it is adopted for graphic representation of images spectrum and prevalent fibers direction which can be highly oriented (elliptic shape) or spread in all directions (circular shape). For each sample four different areas were analyzed.

### 2.15 Gastrocnemius muscles analyses

After implants removal, the gastrocnemius muscles from both the operated and the contralateral sides were harvested by exposing the musculature via a knee to ankle longitudinal skin incision.

Once dissected out from origin to insertion and cleaned, the wet weight (g) was recorded using an electronic balance. The values were expressed as a percentage of the contralateral control to eliminate individual differences [40,41].

### 2.16 Statistical analysis

Statistical analyses were performed using One-way ANOVA and Tukey's multiple comparison test. The results were expressed as mean ± SD. P-values <0.05 were considered as statistically significant. Statistical calculations were made using Prism 9 software (GraphPad Software, San Diego, CA).

## 3 Results

### 3.1 OxPVA-based conduits ultrastructure

**Fig. 1:**
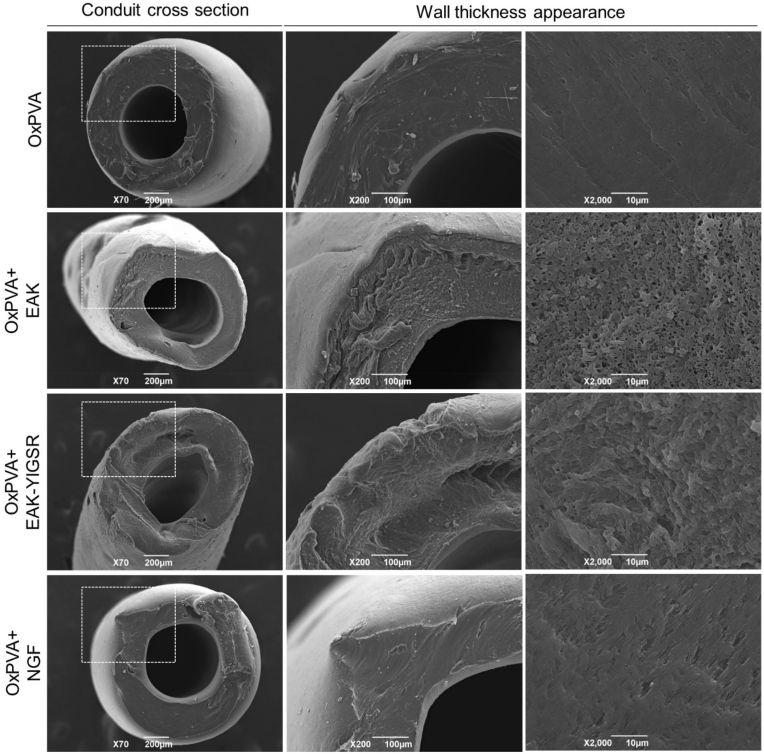
OxPVA-based conduits ultrastructure. SEM photomicrographs showing the conduits cross section and focusing on wall-thickness appearance.

SEM analysis allowed to appreciate OxPVA-based conduits ultrastructure, also referring to the wall thickness appearance. As showed in Fig. 1, a comparable conduits wall section was appreciable among the considered groups; the sections were circular, only slightly altered by cutting or processing artifacts. Conversely, differences were detected in the inner wall. Specifically, at higher magnification, OxPVA was smooth, while a progressive modification characterized by an increased rugosity was detected in OxPVA + NGF, OxPVA + EAK-YIGSR and OxPVA + EAK, respectively.

### 3.2 SAPs homogeneous distribution

**Fig. 2:**
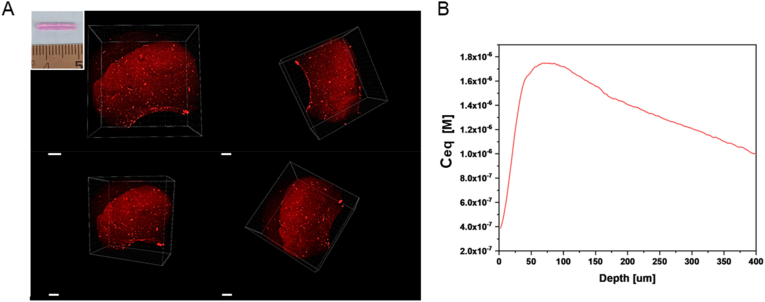
Assessment of efficacy in SAPs mechanical incorporation. A) The EAK rhodamine labelled peptide (EAK-Rh) emits a fluorescent signal collected in the red channel when laser-excitated at 800 nm wavelength in the multiphoton microscope. No signal was detected in the peptide-free scaffold (not reported); the insert in A) shows the macroscopic appearance of a conduit OxPVA + EAK-Rh. Scale bar: 100 μm. B) Calculation of rhodamine (Rh) equivalent concentration (C_eq_) [M] from Rh Intensity (red channel) *versus* depth in the conduits. Maximum concentration: 1.72 × 10^−6^ M. (For interpretation of the references to color in this figure legend, the reader is referred to the Web version of this article.)

Homogeneous distribution of the SAPs within the polymer matrix is fundamental to assure for a fully bioactive device, along its whole lenght; being the first time that OxPVA is mixed with SAPs, this needs to be verified. Once incorporated within the hydrogel, the EAK rhodamine labelled peptide (EAK-Rh) conferred a typical purple colour to the polymer, maintained even after freezing-thawing (FT). Thus, mechanical incorporation efficacy was preliminarily desumed once the conduit was extracted from the mold, appearing as coloured homogeneously (Fig. 2A, insert). Subsequently, this evidence was confirmed by confocal microscopy analysis. As showed in Fig. 2, a uniform fluorescent red/orange-colour signal was detected after the EAK-Rh excitation. In contrast, OxPVA conduits did not emit signal in the same experimental conditions, as expected. Determination of the Rh equivalent concentration (C_eq_) [M], revealed for the EAK-Rh peptide model an average maximum concentration of 1.72 × 10^−6^ M.

### 3.3 Release profile of NGF

The release of NGF from the OxPVA nerve guide was determined by RP-HPLC analysis. The gradient and all the conditions of RP-HPLC, reported in the materials and method section, were the same for the calibration curve runs and for the experimental points. Eight calibration standard concentration levels (excluding the 0-point) were represented in the typical calibration curve shown in supplementary information **(S1).**

After the 24 h NGF adsorption into the OxPVA sample, the quantification of residual NGF in solution was determined through HPLC calibration curve. The same protocol was used to determine the NGF quantity in the two washings after NGF 24 h adsorption step. Considering a single sample pre-treated with 37.5 nmoles of NGF in 300 μL PBS, the residual NGF quantity in solution after 24 h adsorption step was 1.455 nmoles and the NGF in the two following washing resulted in 0.006 nmoles. The data indicated the adsorbed NGF in a single sample to be 36.039 nmoles (96.1% of initial NGF quantity). The release curve is reported in supplementary information **(S2)**.

### 3.4 Tensile testing results

**Fig. 3:**
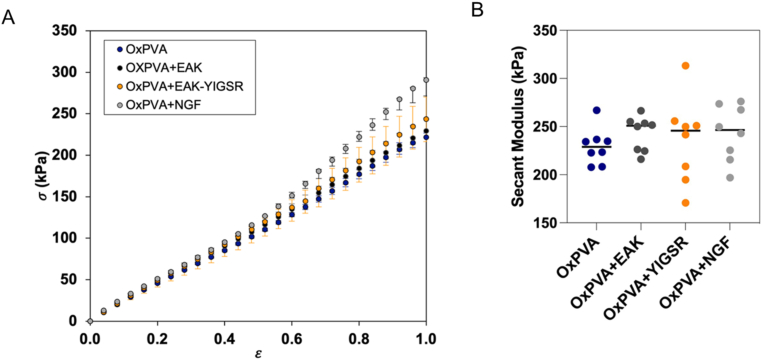
Results of tensile tests on OxPVA hydrogels (i.e., OxPVA, OxPVA + EAK, OxPVA + EAK-YIGSR, OxPVA + NGF): A) Nominal stress σ-ε (median and interquartile range) vs. nominal strain; B) Comparison of secant moduli calculated at 20% strain among all the tested samples. The median of each experimental group is indicated with a black dash. No statistically significant differences were found between the hydrogels.

The possible effect on mechanical properties of the hydrogel after both the incorporation of SAP (i.e., EAK, EAK-YIGSR) and the NGF adsorption was investigated by means of tensile testing. Hence, each sample reached the 100% level of deformation without fracturing. Moreover, all the hydrogels showed an almost linear behaviour; in Fig. 3A, median σ-ε curves with interquartile ranges of each group are represented. To highlight any difference among the hydrogels, secant moduli at 20% strain were calculated (Fig. 3B); no statistical differences were detected, suggesting that both the treatments do not affect the mechanical behaviour of OxPVA hydrogels.

### 3.5 Surgery: nerve injury and grafts implantation

**Fig. 4:**
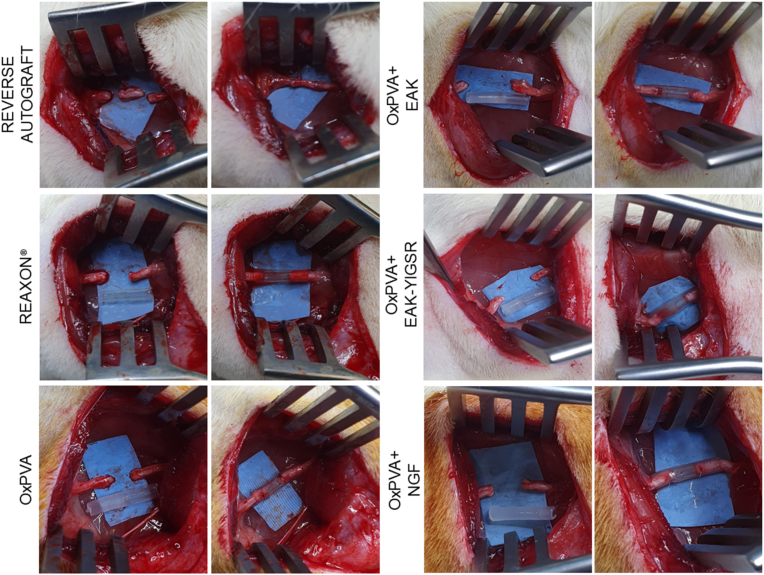
Rats sciatic nerve injury and autograft/nerve conduits implantation (gap size: 5 mm). Autologous nerve inversed graft served as the comparison for the conduits' groups. All the conduits show as transparent connectors; hence, once they were placed, it was possible to verify the correct insertion of the stumps within the devices.

Effectiveness of the nerve conduits needs to be verified in an animal model of PNI, aiming to simulate a common surgical scenario in nerve repair. After sciatic nerve injury, the conduits were sutured to reconnect the proximal and the distal stumps. The OxPVA-based conduits showed to adequately fit and envelope the nerve ends, they were flexible but resistant, without collapsing; no rupture occurred when suturing. Regarding Reaxon®, the devices appeared more rigid than the OxPVA-based counterparts, making more difficult needle penetration. Sometimes a gap could be observed between the epineurium and the inner wall of the conduit, while using the guide with the most adequate size in diameter. All conduits were transparent, thus helping the surgeon in prosthesis positioning (Fig. 4).

### 3.6 Animals wellbeing and nerve function recovery

**Fig. 5:**
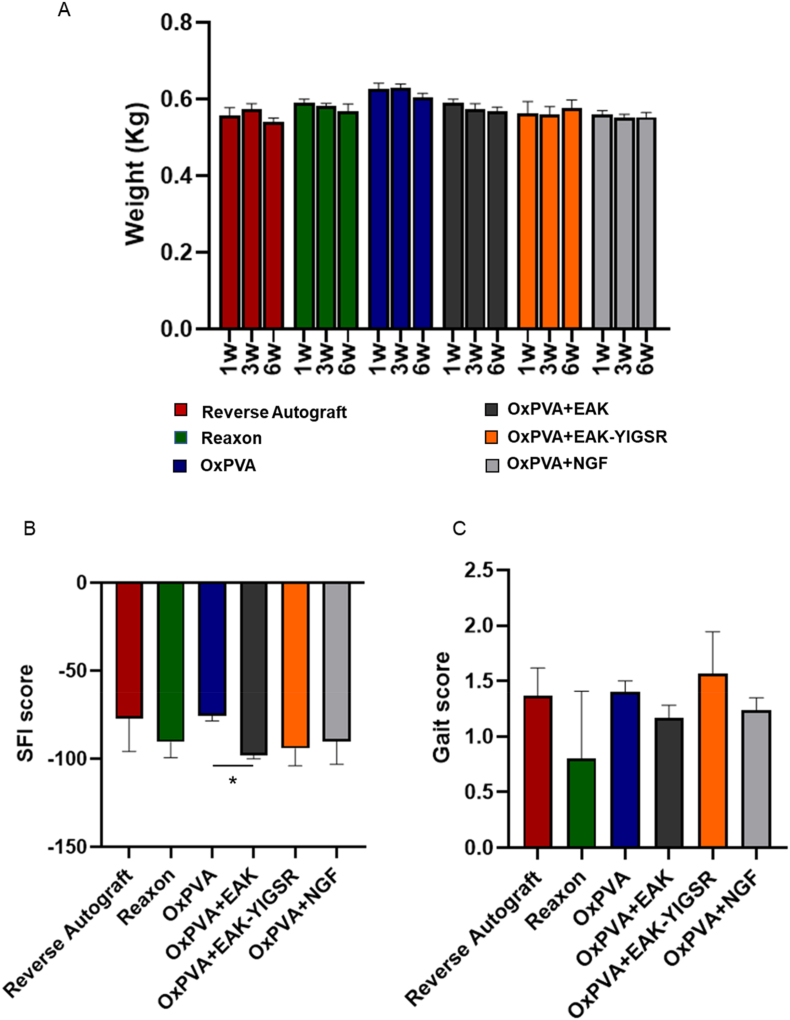
Animals' wellbeing and function recovery assessment. A) Animals weight trend; B) Sciatic Functional Index (SFI) attribution. Score around 0 corresponds to normal nerve function; score around −100 corresponds to total motor sciatic nerve dysfunction; (*p < 0.05). C) Mean score/group for gait analysis (maximum mean score/animal: 2.0). No statistically significant differences were detected comparing the experimental groups in weight, SFI and gait.

Verifying animals’ wellbeing is fundamental, also providing a preliminary information on possible surgery outcome. To this purpose, animal weight was effectively tracked at week 1, 3 and 6 after surgery. No significant weight loss was detected within each experimental group, thus suggesting that all animals well tolerated the surgical procedure (Fig. 5A).

Nerve function recovery was assessed evaluating the animals’ Sciatic Functional Index (SFI) and gait (Fig. 5B and C).

At 6 weeks, the SFI recorded values were −77.20 ± 18.62 for RA; −90.51 ± 8.85 for Reaxon®, −75.62 ± 3.04 for OxPVA, −97.89 ± 2.20 for OxPVA + EAK; −94.14 ± 9.75 for OxPBA + EAK-YIGSR and −90.26 ± 12.90 for OxPVA + NGF. A statistically significant difference (p < 0.05) was only detected between OxPVA and OxPVA + EAK; except for OxPVA-EAK, OxPVA-based conduits showed better results compared to Reaxon® group and similar results compared to the RA (gold standard). Regarding the gait, the overall trend of the graph showed for OxPVA + EAK-YIGSR implanted animals the better outcome within the cohort (1.57 ± 0.38), followed by OxPVA (1.4 ± 0.1), RA (1.4 ± 0.3), OxPVA + NGF (1.2 ± 0.12), OxPVA + EAK (1.57 ± 0.38) and Reaxon® (0.8 ± 0.61); however, no significant differences were evident.

### 3.7 Surgical site evaluation and nerve conduits removal

All animals survived the 6-week period following grafts positioning. Hence, once euthanasia was administered, the first aspect to consider at implants dissection was the macroscopic appearance of the surgical site, possibly correlating with both functional and morpho-structural outcomes.

**Fig. 6:**
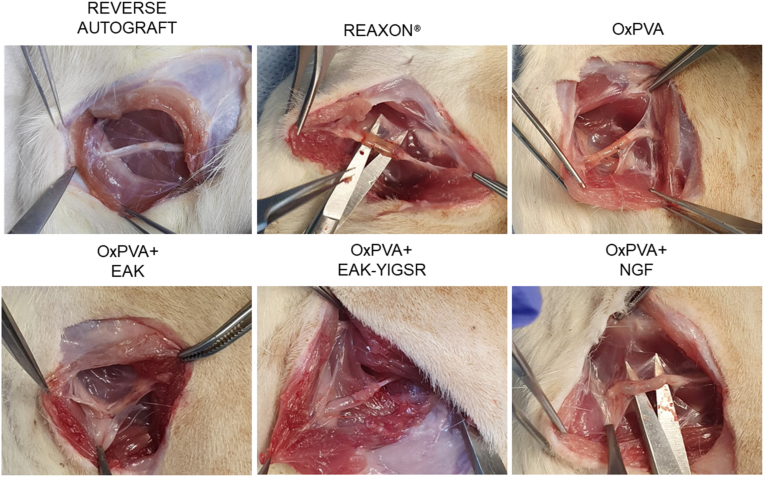
Adherences. Representative images of grafts-associated adherences at 6 weeks from surgery.

At dissection all NCs were clearly recognizable, without dislocation evidence. No signs of inflammation and foreign body reaction were observed in correspondence of the implants and/or in the surrounding tissues; only a thin connective tissue, graded as 2 according to the adhesions score, was recognized around the guides. No differences were evidenced comparing Reaxon® and the OxPVA-based groups *versus* the RA group (Fig. 6).

**Fig. 7:**
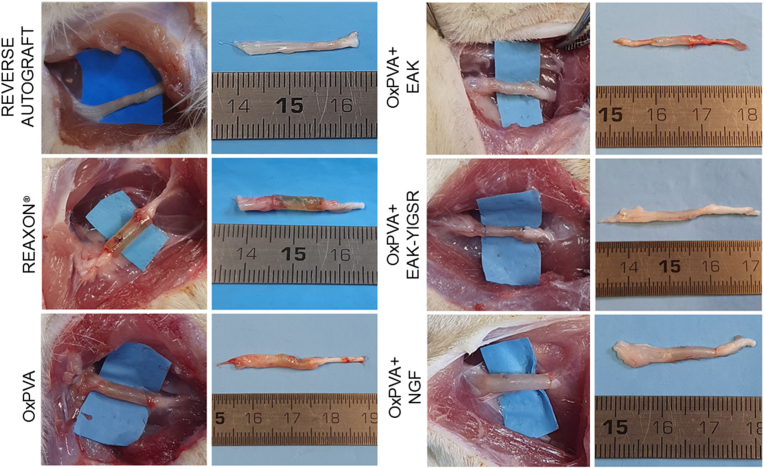
Grafts at 6 weeks from surgery. All implants were identifiable, no dislocation occurred. Guides transparency allowed to detect the presence of material inside.

Once the thin connective sheath was removed, the NCs appeared as firmly anchored to the epineurium and mechanically consistent, thus maintaining their original tubular shape without kinking. Macroscopically, no severe scar tissue formation was detected in correspondence of the suture sites. The gross observation of the devices in situ revealed that the guides were not empty: conduits transparency allowed for this preliminary assessment (Fig. 7).

### 3.8 Explants histological and immunohistochemical characterization

Histological and immunohistochemical characterization of explants allows to preliminarily describe the presence of eventual tissue inside the guides (also discriminating about its specific origin) and possible inflammatory reaction. This type of evaluation can integrate the macroscopic analysis and support subsequent morphometric studies.

**Fig. 8:**
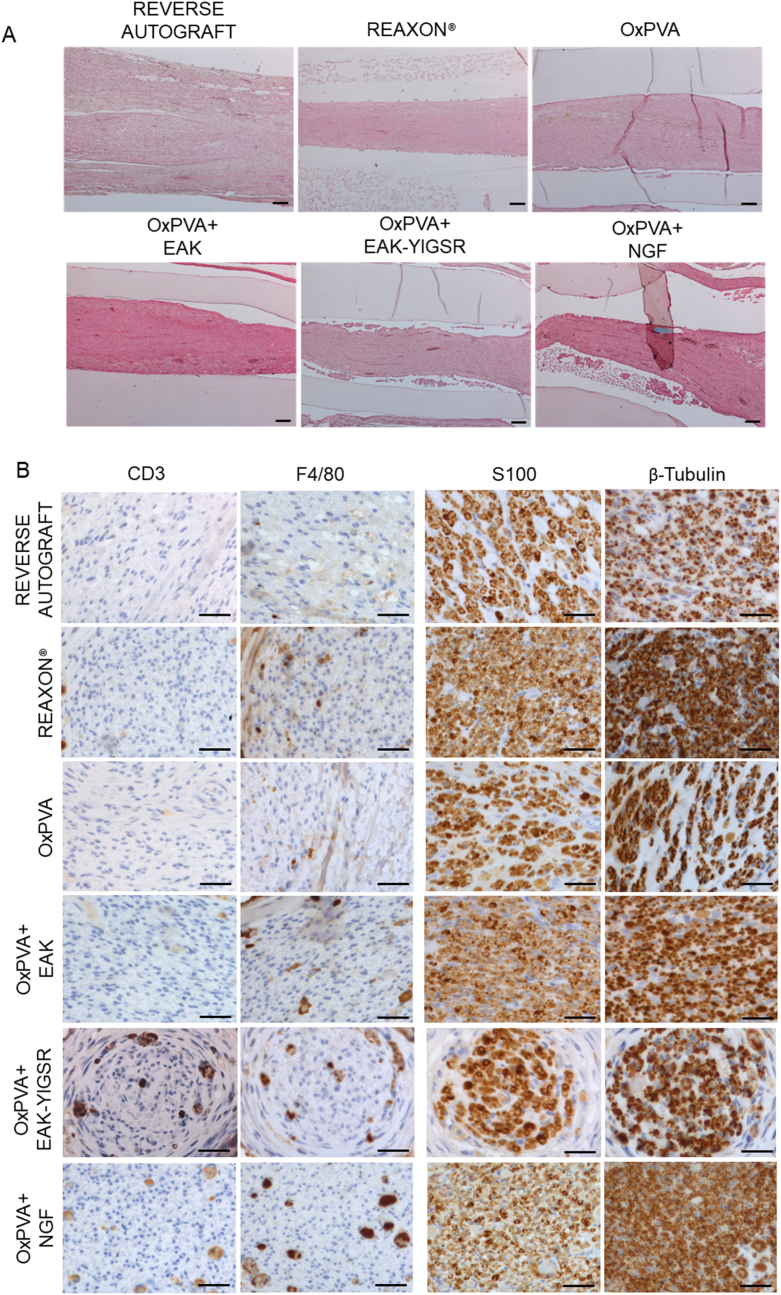
Explants analysis by histology and immunohistochemistry. A) Representative images of longitudinal sciatic nerve sections at 6 weeks from surgery and stained with H&E, middle portion. Regenerated tissue was recognized within all conduits, confirming the preliminary macroscopic evaluation, in situ. Differently from the Reaxon® group, suffering from artefactual events, OxPVA-based implants allow for polymer identification externally. A thick fibro-adipose sheath was observable around the autograft. (Scale bars: 100 μm). B) Immunohistochemical analysis. Anti-CD3, -F4/80, -S100 and -β-tubulin reactions performed on the central portion of harvested implants, at six weeks from surgery (Scale bars: 25 μm).

Once harvested, longitudinal sections of the NCs were stained with haematoxylin and eosin (H&E) and the histological analysis confirmed the presence of regenerated tissue within all the devices; no inflammatory infiltrate was visible. Additionally, in all OxPVA-based specimens, the synthetic polymer was clearly identifiable externally. The hydrogel was smooth, transparent, with an intact texture; no cellular infiltration was observed. Eventual foldings in OxPVA-based NCs were ascribable to cut artefactual events (Fig. 8A). Considering Reaxon®, the commercial conduit was clearly recognizable macroscopically but not in section as it hardly adhered or tolerated the staining procedures.

Biocompatibility of the NCs was confirmed by immunohistochemical analysis (Fig. 8B), thus corroborating H&E preliminary evidence. Transversal sections of the implants middle portion showed the absence of severe inflammatory reactions; eventually, only rare CD3 and F4/80 positive elements were detected. Considering the RA group, as expected, no severe lymphomonocytic infiltration was encountered after surgery, due to the autologous nature of the implant. In parallel, the nervous nature of the regenerated tissue within the conduits was also proved by specific immunohistochemical stainings. Explants middle portions were all immunoreactive for the Schwann cells marker S100 and the axonal marker β-tubulin (Fig. 8B).

### 3.9 Morphometric study

Morphometric evaluation of the samples allows for nerves/axons regeneration process assessment, also highlighting eventual differences among the different devices, acting as a dynamic regenerative chamber.

**Fig. 9:**
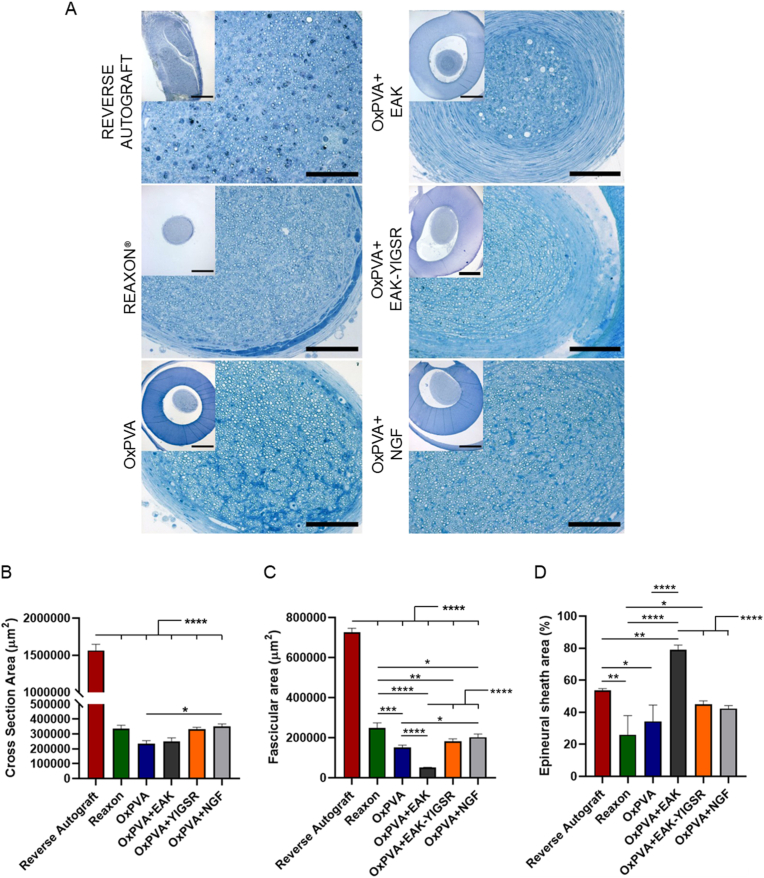
Central portion transversal section morphometry. A) Semithin toluidine blue-stained transversal sections, middle portion. At 6 weeks from surgery, both the epineurium and the OxPVA-based conduits were clearly identifiable, surrounding the regenerated tissue within the guide middle (insert); the photos magnification does not allow to appreciate the Reaxon® conduit (Scale bars: 100 μm; 400 μm (insert)). B-D) Histograms showing mean total cross-section nerve area (μm^2^) (B), fascicular area (μm^2^) (C) and epineural sheath area (%) (D). (*p < 0.05; **p < 0.01; ***p < 0.001; ****p < 0.0001). (For interpretation of the references to color in this figure legend, the reader is referred to the Web version of this article.)

Explants total cross-section area, fascicular area (i.e., only fasci, without the epineurium) and the epineural sheath area percentage at the central portion were preliminary determined on semithin sections after toluidine blue staining (Fig. 9).

Regarding the total cross-section area (μm^2^), that of RA (1564897.46 ± 82321.43) was significantly higher (p < 0.0001) than all the other experimental groups (Reaxon®, 337819.87 ± 20970.40; OxPVA, 233755.74 ± 21984.57; OxPVA + EAK, 248659.32 ± 25336.73; OxPVA + EAK-YIGSR, 333037.19 ± 12355.91; OxPVA + NGF, 351659.01 ± 16103.38). OxPVA + NGF also showed higher values than OxPVA (p < 0.05) (Fig. 9A and B).

Focusing on fascicular area (μm^2^), RA group displayed the highest (p < 0.0001) mean area value within the cohort (RA, 725343.59 ± 20681.78; Reaxon®, 248308.91 ± 25873.38; OxPVA + NGF, 202786.96 ± 15276.12; OxPVA + EAK-YIGSR, 183204.01 ± 11218.59; OxPVA, 151886.44 ± 10805.73; OxPVA + EAK, 51360.06 ± 10805.73); Reaxon® distinguished over OxPVA + EAK (p < 0.0001), OxPVA (p < 0.001), OxPVA + EAK-YIGSR (p < 0.01) and OxPVA + NGF (p < 0.05); whereas OxPVA + NGF showed a mean fascicular area higher than OxPVA + EAK (p < 0.0001) and OxPVA (p < 0.05). OxPVA + EAK-YIGSR associated data were higher than OxPVA + EAK (p < 0.0001) and, finally, OxPVA fasci were dimensionally larger than that measured for OxPVA + EAK (p < 0.0001) and OxPVA + NGF (p < 0.05) groups (Fig. 9A,C).

Fig. 9A allows to appreciate epineural sheath thickness/group; thus, its % was derived. Accordingly, as represented in Fig. 9D, the OxPVA + EAK showed a thicker sheath (79.15 ± 2.82%) than RA (53.61 ± 1.15%)˃OxPVA + EAK-YIGSR (45 ± 2.15%)˃OxPVA + NGF (42.38 ± 1.84%)˃OxPVA (34.39 ± 10.11%)˃Reaxon® (26 ± 12.0%). Multiple comparison analyses higlighted significant differences (p < 0.0001) among OxPVA + EAK *versus* OxPVA-YIGSR, OxPVA + NGF, OxPVA, Reaxon®. Others were detected comparing OxPVA + EAK *versus* RA and RA *versus* Reaxon® (p < 0.01); RA *versus* OxPVA and OxPVA + EAK-YIGSR *versus* Reaxon® (p < 0.05).

**Fig. 10:**
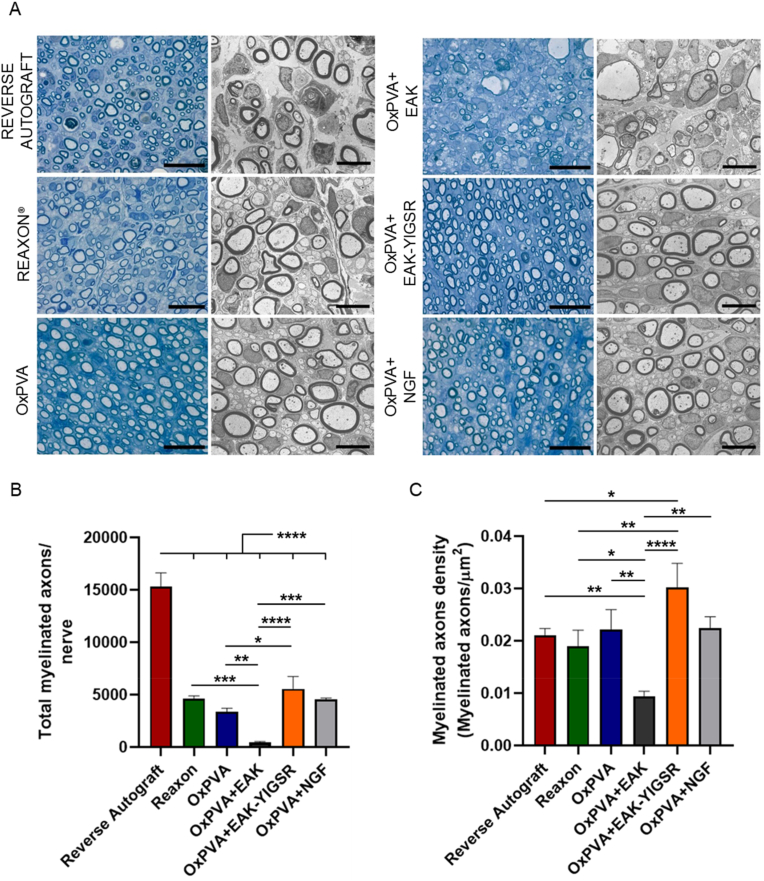
Myelinated axons presence and distribution. A) Evaluation of regenerated tissue quality by toluidine blue staining (left column) and Transmission Electron Microscopy (TEM) (right column) in the middle portion (Scale bar: 20 μm (semithin sections); 6 μm, TEM images). B,C) Histograms show total axons number (B) and axons density (number of axons/fascicular area μm^2^) (C). Results are expressed as mean values ± SD (*p < 0.05; **p < 0.01; ***p < 0.001; ****p < 0.0001). (For interpretation of the references to color in this figure legend, the reader is referred to the Web version of this article.)

Semithin toluidine-blue stained sections and Transmission Electron Microscopy (TEM) images both highlighted homogeneously distributed myelinated and unmyelinated axons in the middle portion of the regenerated nerves (Fig. 10A).

Considering total myelinated axons number/nerve (fasci) (Fig. 10B), RA showed the higher mean value (15313.56 ± 1309.15, p < 0.0001) with respect to all other experimental groups (OxPVA + EAK-YIGSR, 5566.55 ± 1170.46; Reaxon®, 4643.88 ± 251.60; OxPVA + NGF, 4527.11 ± 172.46; OxPVA, 3346.77 ± 359.42; OxPVA + EAK, 480.93 ± 62.23). According to the data analysis, OxPVA + EAK-YIGSR distinguished over OxPVA + EAK (p < 0.0001) and OxPVA (p < 0.05); whereas Reaxon® myelinated axons number was higher than in OxPVA + EAK (p < 0.001). Furtherly, OxPVA + NGF showed higher values with respect to OxPV + EAK (p < 0.001).

As regards the myelinated axons density (axons/μm^2^) (Fig. 10C), the morphometric study revealed this descending order: OxPVA + EAK-YIGSR (0.030 ± 0.004)˃OxPVA + NGF (0.022 ± 0.002)˃OxPVA (0.022 ± 0.004)˃RA (0.021 ± 0.001)˃Reaxon® (0.019 ± 0.003)˃OxPVA + EAK (0.010 ± 0.001), respectively. According to the statistical analysis, OxPVA + EAK-YIGSR showed a mean higher value than OxPVA + EAK (p < 0.0001), Reaxon® (p < 0.01) and RA (p < 0.05). Furtherly, OxPVA distinguished over OxPVA + EAK (p < 0.01); OxPVA + NGF over OxPVA + EAK (p < 0.01) (p < 0.01) and RA over OxPVA + EAK (p < 0.01). Finally, Reaxon® displayed a mean myelinated axons density higher than OxPVA + EAK (p < 0.05).

**Fig. 11:**
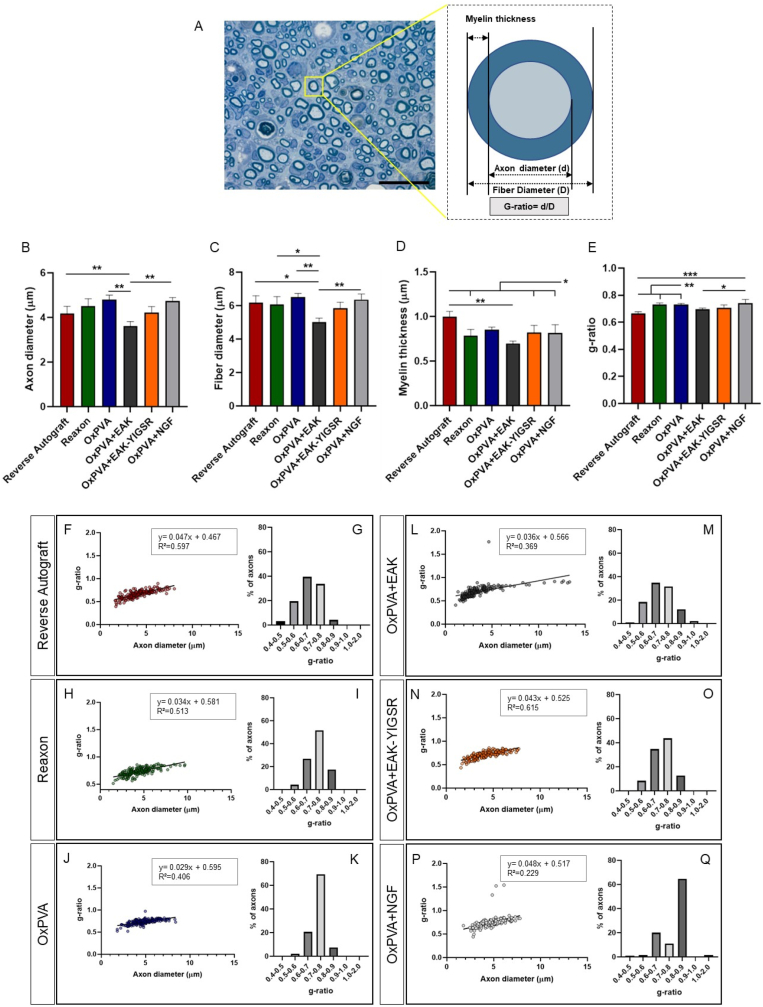
Morphometric analysis of myelinated axons. A) Schematic rapresentation describing g-ratio calculation referred to a rapresentative semithin section (Reverse Autograft central portion, positive control; scale bar: 20 μm). B) Mean axons diameter. C) Mean fiber diameter. D) Mean myelin thickness values. E) Mean g-ratio values calculated for each experimental group. (F,H,J,L,N,P) The g-ratio/axon diameter correlation of individual fibers showing the resulting linear regression lines for each experimental group. (G,I,K,M,O,Q) Assessment of g-ratio values distribution and percentage of prevalence for each experimental group. (*p < 0.05; **p < 0.01; ***p < 0.001).

Mean axons' diameter (μm) was calculated for each experimental group (OxPVA, 4.81 ± 0.20>OxPVA + NGF, 4.73 ± 0.16> Reaxon®, 4.51 ± 0.33>OxPVA + EAK-YIGSR, 4.21 ± 0.27 > RA, 4.18 ± 0.32>OxPVA + EAK, 3.61 ± 0.21); OxPVA, OxPVA + NGF and RA showed significantly higher values (p < 0.01) than OxPVA + EAK (Fig. 11A and B). Contextually, as for the fibers’ diameter (μm) it was observed this trend: OxPVA (6.51 ± 0.22)>OxPVA + NGF (6.37 ± 0.33)>RA (6.17 ± 0.42)>Reaxon® (6.10 ± 0.46)>OxPVA + EAK-YIGSR (5.85 ± 0.36)>OxPVA + EAK (5.00 ± 0.25). According to the statistical analysis, OxPVA and OxPVA + NGF distinguished over OxPVA + EAK (p < 0.01); RA showed higher values than OxPVA + EAK (p < 0.05) as well as Reaxon® over OxPVA + EAK (p < 0.05) (Fig. 11C).

Myelin sheath was measured (μm) and a progressive reduction in thickness was recognized as described onward: RA (0.997 ± 0.069)>OxPVA (0.853 ± 0.027)>OxPVA + EAK-YIGSR (0.819 ± 0.079)>OxPVA + NGF (0.817 ± 0.092)>Reaxon® (0.783 ± 0.071)>OxPVA + EAK (0.710 ± 0.026). RA showed higher values than Reaxon®, OxPVA + EAK-YIGSR and OxPVA + NGF (p < 0.05) as well as compared to OxPVA + EAK (p < 0.01) (Fig. 11D).

Moreover, the mean g-ratio (axon diameter/fiber diameter) was preliminarily focused (RA, 0.665 ± 0.012; Reaxon®, 0.732 ± 0.011; OxPVA, 0.732 ± 0.01; OxPVA + EAK, 0.697 ± 0.01; OxPVA + EAK-YIGSR, 0.708 ± 0.020; OxPVA + NGF, 0.743 ± 0.026). OxPVA + NGF distinguished over both RA (p < 0.0001) and OxPVA + EAK (p < 0.05) while OxPVA and Reaxon® over the RA group (p < 0.01) (Fig. 11E). Thus, g-ratio/axon diameter correlation of individual fibers was determined (Fig. 11F,H,J,L,N,P). As expected, g-ratio increased slightly with axon diameter, suggesting that myelin thickness relative to axon diameter decreased at larger axon diameters. Hence, the linear regression lines were calculated. Within the study cohort, similar R-squared (R^2^) values were observed for RA (R^2^ = 0.597) and OxPVA + EAK-YIGSR (R^2^ = 0.615). The g-ratio ranges distribution/prevalence was reported as percentage; the intervals 0.6–0.7 and 0.7–0.8 were the most represented in all the experimental groups, except for the OxPVA-NGF group (main g-ratio interval: 0.8–0.9) (Fig. 11G,I,K,M,O,Q).

### 3.10 Second Harmonic Generation Microscopy: intensity and coherency

**Fig. 12:**
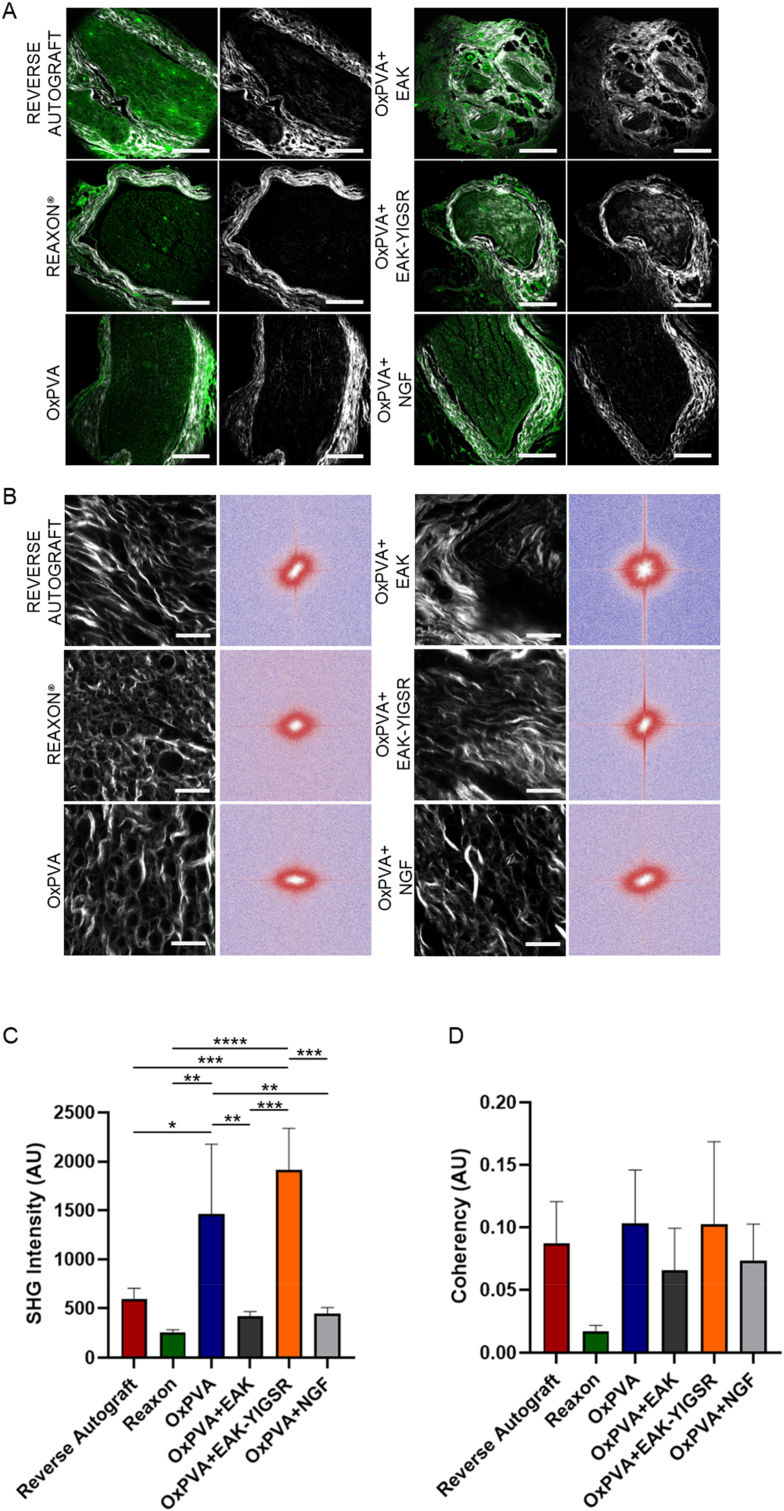
Fibroconnective tissue evaluation. A) Second Harmonic Generation (SHG) Microscopy of repaired sciatic nerves at 6 weeks after surgery. Representative cross sections of the harvested samples central portions, qualitatively evaluated by SHG microscopy: in green the autofluorescence signal; in white, collagen's SHG signal at 400 nm (scale bars: 200 μm). B) SHG microscopy images (left column) and Fast Fourier Transforms (FFTs) (right column) applied to samples, showing a circular and an oval collagens orientation within the regenerated fasci. C) SHG intensity evaluation (*p < 0.05; **p < 0.01; ***p < 0.001; ****p < 0.0001). D) Coherency analysis describing collagen orientation: coherency is bounded between 0 and 1, with 1 indicating highly oriented structures and 0 indicating isotropic areas. No significant differences were detected within the study cohort. (For interpretation of the references to color in this figure legend, the reader is referred to the Web version of this article.)

Eventual structural and inflammatory responses elicited by different NCs implantation were assessed by Second Harmonic Generation (SHG) microscopy. The presence of the fibrous epineural tissue was evident in all the samples; it appeared more compact in Reaxon® and OxPVA, while laxer (even if clearly recognizable) in the other experimental groups. In RA, OxPVA + EAK and OxPVA + EAK-YIGSR it was also identifiable around fasci, constituting the perinevrium (Fig. 12A). Furtherly, a certain amount of fibroconnective tissue was recognized in the middle of the nerves’ sections, dispersed among the functional tissue: Fast Fourier Transform (FFT) analysis allowed to visualize fibers orientation (Fig. 12B).

The intensity of the SHG signal was quantified within the cohort (Fig. 12C): OxPVA + YIGSR (1915 ± 366 arbitrary unit, AU) and OxPVA (1461 ± 319 AU) samples distinguished for higher collagen infiltration with respect to the others experimental groups, according to the following order: RA (593 ± 98 AU); OxPVA + NGF (444 ± 56 AU); OxPVA + EAK (417 ± 43 AU); Reaxon® (254 ± 27 AU). Significant differences were detected comparing: OxPVA + EAK-YIGSR *versus*: Reaxon® (p < 0.0001), OxPVA + NGF, OxPVA-EAK and RA (p < 0.001); OxPVA *versus* OxPVA + EAK, OxPVA + NGF and Reaxon® (p < 0.01); OxPVA *versus* RA (p < 0.05). SHG microscopy intensity signal was than correlated to SGH coherency (Fig. 12D). Interestingly, despite both OxPVA + YIGSR and OxPVA samples displayed a higher collagen content, the fibers tended to be mainly orientated in one direction (anisotropic behaviour), suggesting a directional disposition coherent with a nervous regenerative process inside nerve conduits. No significant differences were detected in Coherency.

### 3.11 Gastrocnemius muscle evaluation

**Fig. 13:**
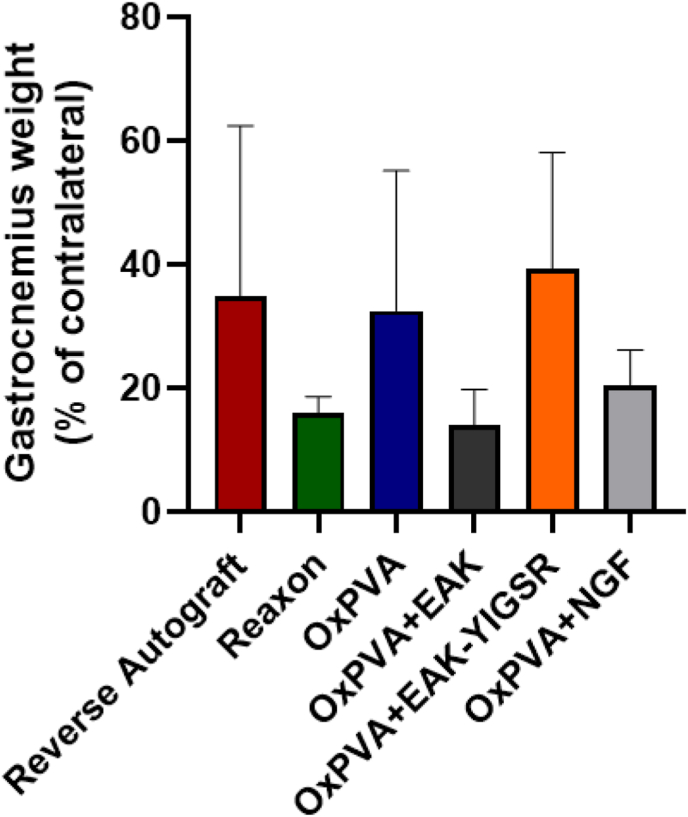
Gastrocnemius muscle. Gastrocnemius weight percentage (%): values are expressed as percentage of the contralateral, unoperated limb (mean values ± SD). No significant differences were detected within the study cohort.

After sciatic nerve transection, long-term denervation of the gastrocnemius muscle leads to its atrophy, typically characterized by wet weight reduction. Hence, the gastrocnemius muscles wet weights % (operated limb side *versus* contralateral side) were determined after 6 weeks from surgery and grafts implantation. Despite no significant differences comparing the experimental groups, the calculated % values followed this descending order OxPVA + EAK-YIGSR (39.25 ± 18.84%)>RA (34.95 ± 27.48%)>OxPVA (32.47 ± 22.69%)> OxPVA + NGF (20.34 ± 5.84%)>Reaxon® (15.92 ± 2.72%)>OxPVA + EAK (14.11 ± 5.67%) (Fig. 13).

## 4 Discussion

In case of severe 10.13039/100008337PNI, peripheral nerves are endowed with a certain ability to regenerate; however, to efficiently support this process, both an optimal and “on-time” surgical reconstruction is required [42]. Within this clinical context, to overcome autograft positioning limitations, the identification of biocompatible, biodegradable synthetic polymers allowing for the fabrication of advanced and tunable NCs is particularly intriguing. In the wide panorama of biomaterials, the new OxPVA stands out revealing as a promising resource for smart prosthesis fabrication in tissue engineering [2,[16], [17], [18],^20^,^29^,^43^]. However, despite first appealing *in vivo* results, its specific chemical and physical characteristics still allow for intense research and development activities [22,23,43,44] to assure a fine control over pro-regenerative behaviour of the derived devices.

When developing devices for PNI recovery, to establish a nerve growth-promoting milieu is an ambitious goal to aim for. Vanguard NCs are not merely pipes fabricated to connect the opposing stumps of an injured nerve; they are required to be highly bioactive in turn boosting axons’ number and regeneration speed and length, while discouraging fibroconnective tissue formation/infiltration [29]. To this purpose, addition of signal moieties/factors can be adopted [27] and represents the approach followed here for OxPVA-based NCs improvement. As broadly recognized [45], the three-dimensional hydrogels matrix made of cross-linked hydrophilic polymer chains can act as a promising carrier for molecules; entrapment, adsorption, and covalent attachment are the possible immobilization strategies to resort to Ref. [46]. Specifically, within this study, different neuro-instructive sequences were compared: EAK, EAK-YIGSR, NGF.

Considering *in vivo* biocompatibility as the fundamental critical issue to face with during immobilization, EAK and EAK-YIGSR entrapment and NGF adsorption were preferred, eluding the employment of chemical agents (reducing agents) for stable NCs decoration with molecules. According to our knowledge, mechanical incorporation of the SAPs within a polymeric matrix is a new strategy for scaffolds bioactivation; in particular, focusing on NCs, the SAPs have been commonly suggested as lumen-filling materials only [24], not as trappable sequences. Following first encouraging in vitro evidences on EAK mechanical incorporation [29], the *in vivo* outcome descending from this strategy was studied, preliminarily confirming the method efficacy (EAK-Rh homogeneous distribution) on NCs samples models by confocal microscopy. In parallel, *in vivo* results from mild/weak interactions between a neuroactive cue (adsorbed NGF) and OxPVA NCs were also assessed. NGF, which is physiologically produced by Schwann cells within the nerve, has a potential positive role in myelination; whereas, OxPVA attitude to behave like a drug delivery system was well described in the past (coupling with Ciliary Neurotrophic Factor (CNTF) and CNTF linked to the *trans*-activator transduction domain (TAT, TAT-CNTF)) [19,21] and here confirmed also for NGF. An effective loading of the growth factor was observed (96.1% of initial NGF quantity), in the face of a progressive release in the time interval considered. Furthermore, it must be considered that, *in vivo*, the dynamic mechanical environment in delivery site influences the growth factor release rate; thus, mechanical loading can drive the bioactive molecules delivery [47].

In this study, SAPs and NGF bioactivated conduits (OxPVA + EAK, OxPVA + EAK-IKVAV, OxPVA + NGF) were compared for preclinical outcomes *versus* RA, OxPVA and Reaxon® devices. RA mimics the gold standard autograft (positive control); OxPVA alone is known to be effective in supporting peripheral nerve regeneration [20] and previously also distinguished as an interesting alternative to the expensive commercial product Neurawrap™ [2]; chitosan-based nerve grafts stood out for excellent biocompatibility, permeability, plasticity and biodegradability leading to satisfactory clinical results. Furtherly, the chitosan degradation products can boost 10.13039/100008337PNI recovery, supporting and ameliorating the development of macrophage-constructed microenvironments [48].

Animal models are fundamental for new advancements in PNI research; in particular, due to its realistic testing bench capability, rat sciatic nerve is the gold standard for the studies considering peripheral nerves regeneration by NCs/nerve grafts [49,50]. At the time of conduits positioning, the OxPVA-based devices were easily manipulable, also showing an interesting elastic behaviour. Differently from the more rigid Reaxon® conduits, often displaying a gap between the stump and the conduit after placement, the OxPVA ones were well-fitting, adapting to different native nerves calibres and possibly responding to inter-individual variability. This is an appealing behaviour, even compared to autografts whose choice is necessarily bound to the donor and host nerves diameters (ideally, for good outcomes, an exact correlation is expected between the nerve graft and the host nerve ends) [51]. Moreover, all conduits were resistant to suture without giving rise to ruptures, even in presence of the SAPs. In fact, the presence of bioactive molecules showed to slightly alter the inner wall appearance, possibly due to micro-air bubbles incorporation and/or bioactive molecules interaction with the hydrogel net, while not altering the mechanical behaviour of the OxPVA-based materials at typical physiological strain level (i.e., 20%) [52], as showed by the secant moduli calculated at this latter deformation.

During the evaluation period (six weeks), the animals showed to have tolerated the surgery well, as no significant differences were recognized in weight comparison along time. Then, functional recovery, descending from adequate distal reconnections, was verified to assess neuroregeneration level. The rat sciatic nerve innervates the hindlimb through the peroneal, tibial, and sural branches; these, at last, end in different extensors and flexors along the tibia, foot, and toe. Altering sciatic nerve continuity compromises lower leg function (i.e., locomotion, walking pattern, toe positioning, hindlimb strength) [53].

Within the study cohort, the best SFI results were displayed by OxPVA implanted animals, followed by the RA ones. Differently, presence of EAK or EAK-YIGSR was not associated to a particularly outstanding outcome, similarly to OxPVA + NGF and Reaxon®. As for the gait, a couple of deviations were identified here compared to the SFI results. Despite no significant differences in the mean assigned scores, Reaxon® group displayed the lower performances with respect to the functional parameters focused; while, the presence of the EAK-YIGSR SAP was related to satisfactory outcomes, even (slightly) better to that guaranteed by RA. OxPVA confirmed as an intriguing device, showing a mean gait score superimposable to that of the gold-standard RA; comparable results were observed for OxPVA + EAK and OxPVA + NGF groups which, similarly for the SFI, were worse than RA and OxPVA groups.

Differently from humans, functional recovery is difficult to measure in animals [54]. For long time, determining the SFI has been believed a reliable method from the 3rd week on, after a severe lesion of the sciatic nerve in rats [55,56]. However, in cases showing flexion contractures resulting in print smearing and tail dragging, the utility of SFI is limited [33,54,57,58]; additionally, the speed of gait can be seldom a confounding factor for accurate parameters assessment [40]. Evaluating rat sciatic nerve function recovery after lesion is still a challenge. All traditional tests have limitations and results may not always correspond to function restoration. Hence, a combination of strategies needs to be applied for a more global assessment of nerve function as no one test alone is sufficient [40]. In consideration of this, gastrocnemius weight was registered to integrate SFI and gait analyses. Gastrocnemius is supplied by the posterior tibial branch of the sciatic nerve; denervation effects appear suddenly whereas recovery is slow, indicating reinnervation. Like for gait analysis results, better outcomes were associated to OxPVA + EAK-YIGSR prosthesis whereas the OxPVA + EAK implants were not particularly outstanding. According to the literature, the laminin-derived peptide YIGSR distinguishes for its ability to act on the growth cone or Schwann cells promoting their attachment and migration *in vivo* [59,60]. Furtherly, the present study evidences may also suggest its contribution in guiding regenerating axons in appropriate targets reaching. The fundamental role exerted by YIGSR *in vivo* is certainly corroborated by OxPVA-EAK conduits functional data: EAK alone displayed for less encouraging results, even lower than peptide/growth factors-free OxPVA, in turn comparable with RA.

Once euthanized, the animals were dissected for samples retrieval. Preliminarily, evaluation of the surgical site confirmed the biocompatibility of all the devices: inflammation-related signs were absent, and all the conduits were clearly identifiable, with no dense scar tissue at the outer area of the implant. Extraneural scarring can be established during the wound healing process, whether excessive it can alter an adequate nerve function recovery; all the nerve conduits discouraged this event effectively. Additionally, the guides remained in situ for the considered period without traslocation signs. This correlates with a correct positioning, allowed by conduits transparency but also suggests the adequacy of the cross-linking protocol (FT) for the OxPVA-based devices (not altered by the introduction of the SAPs). Overall, the implants well tolerated the mechanical stress ascribable to animals’ limb mobility.

Together with supporting surgical repair, the guides transparency also allowed for regenerated tissue detection inside, elongating from the proximal to the distal ends. This is a great advantage allowing for direct detection of nerve fibers in situ, without the need to cut open the conduits for the observation of regenerative nerve [61,62]. Schwann cells are crucial for axonal branching and myelin production [63]; thus, Schwann cells reactivity and cytoskeleton organization were assessed by S100 and β-tubulin immunostaining. Histopathological evaluation confirmed the presence of nervous tissue inside the conduits, without severe inflammatory cells infiltration (lymphocytes, CD3; macrophages, F4/80) and reactions (intraneural scarring).

Collagen production and remodeling are key stages in guiding an injured tissue healing [64]. With a focus over peripheral nerve injury, collagen structure (internal and external) is crucial to guarantee for a proper nerve function during both normal limb movement and nerve regeneration after injury [65]. As highlighted by SHG microscopy, a reliable and non-destructive method for imaging collagen in tissues [66] and a valuable tool for imaging nerves microanatomy [67], fibroconnective bundles were identifiable with differences within the study cohort. Signal intensity (suggesting fibers thickness and/or prevalence) and fibers orientation resulted compatible with both the injury type/severity and reparation strategy. In fact, as a 10% strain is sufficient to impart a loss of internal fibers linearity with progressive damage accumulation and functional deficit [65], nerve continuity interruption may be likely associated with a more critical scenario.

Nerve regeneration supported by OxPVA + EAK-YIGSR and OxPVA displayed the most intense SHG signals within the study cohort in contrast with Reaxon®, responsible of a signal intensity even lower than RA (not statistically significant); OxPVA + NGF and OxPVA + EAK were both comparable to Reaxon® and RA. Collagen production after nerve injury can be affected by a plethora of events (e.g., cell signalling factors release, molecular interactions in the local regenerative milieu) and surgical repair is primarily responsible for triggering and exacerbate this reaction as consequence of sutures (foreign body), possible tension at the repair site and vascular insult following nerve dissection [64]. According to these data, it could be hypothesized an adequate injury recovery after all conduits/graft positioning, except for OxPVA + EAK-YIGSR and OxPVA. However, fibers orientation needs to be also considered, as their alignment is important to provide the regenerated nerve with appropriate function, descending from organized axons growth [68]. Interestingly, while displaying the higher values in collagen signal intensity, OxPVA + EAK-YIGSR and OxPVA both showed anisotropically oriented fibrils, resembling that found in the RA graft (gold standard). Differently, an isotropic (more disorganized) behaviour was observed for tissues within the Reaxon® conduits.

Morphometric study was finally performed. After considering cross section area, fascicular area and epineural sheath area, the parameters of the nerve fiber population were assessed. Total myelinated axons number and density, evaluation of mean axon diameter, myelin sheath thickness and g-ratio represent paramount metrics for clinicians and researchers, frequently used for diagnosis and staging of neuropathic diseases and therefore commonly accepted as outcome measures in nerve regeneration studies [69]. Animals implanted with RA showed the highest mid cross section area within the cohort (p < 0.0001), as expected; whereas all other groups were comparable in this regard (only a significant difference between OxPVA + NGF versus OxPVA, p < 0.05), suggesting a similar regeneration speed. According to the literature, proximal and distal nerve stumps have a growth rate of 0.32 and 0.18 mm/day, respectively, in rat sciatic nerves [70]. Thus, stumps reconnection across a 5 mm gap may occur in 10 days. Comparing transversal sections, differences were then observed in the epineural sheath: OxPVA + EAK distinguished for a remarkably fibrous circumferential epineurium, reducing the fascicular area and, consequently, the number of total myelinated axons/nerve and myelinated axons density. This may in turn reflect over functional recovery as supported by the less encouraging results for this group within the study cohort. Possibly, EAK may activate epineurium cells (i.e., fibroblast) in turn reacting by a higher proliferation rate. No significant differences were detected in epineurium between the positive control RA *versus* OxPVA + EAK-YIGSR and OxPVA + NGF. The thinner sheath was observed for Reaxon® implanted animals, appearing as not significantly different than OxPVA and OxPVA + NGF.

Total axons number was significantly higher in the RA group (p < 0.0001) due to grafts intrinsic characteristics. Whereas, among the conduits, OxPVA + YIGSR distinguished for the better outcomes in supporting regeneration of axons, comparable in mean total number with that of OxPVA + NGF and Reaxon®. Intriguingly, also in terms of myelinated axons’ density, OxPVA + EAK-YIGSR stood out, with results even higher than that displayed by RA (p < 0.05) and similar to that triggered by OxPVA + NGF and OxPVA devices. These analyses confirm the YIGSR role in boosting the sciatic nerve regeneration *in vivo* [71]. In particular, considering the results gathered from OxPVA alone, it may be assumed that EAK-YIGSR furtherly improves the already good performance of OxPVA [17,20]. Despite EAK-YIGSR intrinsic bioactivity, the SAP contribution to hydrogel ultrastructural organization cannot be excluded, supplying to the establishment of a favorable environment for nerve regeneration within the conduit [23,72]. For comparison, the normal axons density in uninjured Sprague Dawley sciatic nerve is 0.0223 ± 0.0022 (/μm^2^) [73]: this data is nearly superimposable with that gathered here for OxPVA + NGF (0.022 ± 0.002), OxPVA (0.022 ± 0.004), RA (0.021 ± 0.001) and Reaxon® (0.019 ± 0.003); slightly lower and higher than OxPVA + EAK-YIGSR (0.030 ± 0.004) and OxPVA + EAK (0.010 ± 0.001), respectively.

The myelin sheath insulates axons, thereby preserving their integrity and promoting neuronal signal transduction [74]: an increase in axonal diameter sustains thickening of its myelin sheath with consequent elongation of the internodal segment and increase in the saltatory conduction velocity [75]. Different nerve fibers diameters imply different conduction speeds; typically, the larger the diameter of a nerve fiber (axon + myelin), the faster it will conduct. Moreover, the diameter distribution of myelinated axons can reflect the quality of regenerated neural tissue. The ratio of the axon diameter to the total diameter (g-ratio) is closely related to the conduction velocity and represents an index suggesting regenerated myelinated nerve fibers maturity and a fundamental tool to characterize nerve fibers in “healthy”. As stated by Rushton [76], a g-ratio value of 0.6 is optimal for the conduction velocity of nerve impulses; however, also deviations from that threshold need to be also interpreted. In accordance with Thomas and Ochoa [77], g-ratio values below than 0.4 may suggest presence of degenerated nerve fibers with abnormal myelin sheath thickening, whereas values higher than 0.7 suggest either regenerated fibres with thinner myelin sheath, or demyelinated nerve fibers. Still today, g-ratio and diameter remain significant tools for the analysis of the myelinated nerve fibers in ‘‘healthy’’ peripheral nerves as well as in different types of neuropathies. Considering the g-ratio interval 0.5–0.7, RA showed here a prevalence of 58.95%; a similar appearance was displayed by OxPVA + EAK group (53.16%), followed by OxPVA + EAK-YIGSR (43.15%). Differently, lower percentages were calculated for Reaxon®, OxPVA and OxPVA + NGF (31.05%; 22.75%; 21.69%, respectively). Beyond this comparison, while g-ratio below 0.4 were not represented within the cohort, deviations higher than ± 0.1 from the “ideal” 0.6 threshold should be carefully assessed and interpreted.

Only a broad overview over implants performance, based on different methods, can guide towards the identification of the most promising prosthesis for a successful peripheral nerve regeneration; thus, we believe that both functional evidences and morphometric data must be collected and integrated each other, to provide for a broad and as much as complete as possible characterization profile of the devices. Here, although the OxPVA + EAK-YIGSR group stands out for its myelinated axons total number and density, the statistical analysis for the SFI study highlighted no significant differences comparing all OxPVA-based conduits mean values with that of the gold standard RA (only a p < 0.05 was detected between OxPVA + EAK and OxPVA; better outcome for this latter). Similarly, according to the gait analysis, all groups were comparable in the gait score and no significant difference aroused. Possibly, a 6 week period may not be sufficient to assess motor recovery in sciatic nerve defect model: a period longer would be useful and this may represent a study limitation.

Focusing on the YIGSR sequence and the growth factor NGF, being biological cues, they are expected to promote axons regeneration [78]; as all the bioactivated nerve conduits are made of OxPVA, the effect of the bioactive molecules can be inferred by comparison with the OxPVA group.

Certainly, electrophysiological evaluation together with data on a sham control group (here not provided and possibly representing a study limit) and outcomes analysis after a longer period of *in vivo* implant (also referring to OxPVA-based conduits attitude in terms of biodegradation), may be of paramount importance to assess motor recovery in sciatic nerve defect model and for a more conscious interpretation of the devices behaviour and the current results.

## 5 Conclusions

All devices supported nerve regeneration without triggering inflammatory reactions and extraneural scarring. According to the morphometric study results, OxPVA nerve conduits were re-confirmed as interesting prosthesis for nerve injury repair [20]. The regenerated tissue showed a total myelinated axons number/nerve and a myelinated axons density similar to that observed for the Reaxon®. Similarly, also for axons and fibers diameter as well as myelin sheath thickness they were comparable, with a prevalent g-ratio distribution within the interval 0.7–0.8. OxPVA + EAK was the less promising conduit of the cohort. Despite the axons showed a g-ratio trend comparable to that of autograft (g-ratio: 0.6–0.7 > 0.7–0.8 > 0.5–0.6 > 0.8–0.9), experimental evidences suggested that EAK peptide incorporation may act as a trigger for a certain fibrotic response outside the axon, not evidenced neither for OxPVA nor for OxPVA + EAK-YIGSR. Regenerated nerves within the OxPVA + EAK conduits showed the thicker epineural sheath area and the smallest fascicular area; hence, it could be speculated that fibroblasts (the cell type of the epineurium) are hyper-activated by the sequence and such activation results in their subsequent proliferation. Additionally, lower values in total myelinated axons number and density were also displayed by this group. Intriguingly, the addition of YIGSR sequence to EAK reversed the mophostructural outcome of the regenerated tissue compared to that sustained by OxPVA-EAK. The experimental evidences highlighted OxPVA + EAK-YIGSR as the most promising prosthesis compared to the others here assayed. Total myelinated axons number (similar to that of Reaxon®) and density were higher than that observed for the other devices; axon diameter and fiber diameter were pretty equal to that showed by RA and Reaxon® while myelin thickness was superimposable than that observed for the commercial group. The g-ratio distribution was mainly represented within the interval 0.7–0.8 suggesting the presence of slightly thicker axons with a thinner myelin sheath than OxPVA-EAK. The addition of EAK-YIGSR to OxPVA appeared as particularly interesting to boost OxPVA intrinsic behaviour; a favorable modification of polymer chains organization together with the specific neurotrophic characteristics of the sequence may represents the key elements of the device success. Further studies considering longer evaluation periods may be accomplished. Finally, OxPVA + NGF showed morphometric results (fascicular area, epineural sheath, total myelinated axons and myelinated axons density) that were similar to that triggered by OxPVA. While a not effective functionalization may initially be hypothesized, differences in g-ratio distribution (higher prevalence of thick fibers with a thin myelin) suggested for OxPVA + NGF conduits a drug-delivery system-like behaviour.

Reinnervation and regeneration are both essential for function restoration after surgical nerve repair [64] and, as firmly stated by Dahlin et al. [79], a proper environment can undoubtedly encourage axonal growth thus supporting the intrinsic growth capacity of peripheral nerve. OxPVA confirmed as a versatile polymer for applications in peripheral nerve regeneration, in particular the functionalization with the SAP EAK-YIGSR through mechanical incorporation appeared as highly appealing. According to our knowledge, this is the first effectiveness verification *in vivo* of such hybrid composite (synthetic polymer + SAP). In future, considering the intrinsic appealing characteristics of both the OxPVA and the SAPs, several studies may be developed: dual-site functionalized of OxPVA platform could be fabricated to boost regeneration [80] and/or control inflammation following injury [81]; moreover, it will be interesting to investigate the possibility to use the EAK-YIGSR as a luminal filler: together with the biochemical bioactivation, a physical stimulus due to the specific ultrastructural organization of the peptide in an aqueous medium (typical of SAPs) may optimize the performances of the OxPVA-based devices guiding the targeted axons regeneration within the conduits.

## Funding

This work was supported by “Consorzio per la Ricerca Sanitaria” (CORIS) of the 10.13039/501100009878Veneto Region, Italy (L.i.f.e.L.a.b. Program). Grant number DGR1017 (July 17, 2018).

## CRediT authorship contribution statement

**Elena Stocco:** Conceptualization, Methodology, Investigation, Formal analysis, Data curation, Writing – original draft. **Silvia Barbon:** Investigation, Data curation, Writing – review & editing. **Diego Faccio:** Investigation. **Lucia Petrelli:** Investigation. **Damiana Incendi:** Investigation. **Annj Zamuner:** Investigation. **Enrico De Rose:** Investigation. **Marta Confalonieri:** Investigation, Writing – original draft. **Francesco Tolomei:** Investigation. **Silvia Todros:** Methodology, Investigation, Supervision. **Cesare Tiengo:** Investigation, Methodology. **Veronica Macchi:** Methodology, Supervision, Writing – review & editing. **Monica Dettin:** Methodology, Data curation, Supervision, Writing – review & editing. **Raffaele De Caro:** Conceptualization, Supervision, Writing – review & editing. **Andrea Porzionato:** Conceptualization, Methodology, Supervision, Writing – review & editing, Funding acquisition.

## Declaration of competing interest

The authors declare that they have no known competing financial interests or personal relationships that could have appeared to influence the work reported in this paper.

## Data Availability

Data will be made available on request.
