# Supplementary material for: Development and preclinical evaluation of bioactive nerve conduits for peripheral nerve regeneration: A comparative study
Source: Mater Today Bio. 2023 Aug 5;22:100761. doi: 10.1016/j.mtbio.2023.100761 (PMC10433238; doi:10.1016/j.mtbio.2023.100761)
Supplement: Multimedia component 1 [file mmc1.docx]

**Development and preclinical evaluation of bioactive nerve conduits for peripheral nerve regeneration: a comparative study**

Elena Stocco^1,2,3^, Silvia Barbon^1,3^, Diego Faccio^4^, Lucia Petrelli^1^, Damiana Incendi^1^, Annj Zamuner^3,5,6^, Enrico De Rose^1^, Marta Confalonieri^1,6^, Francesco Tolomei^6^, Silvia Todros^6^, Cesare Tiengo^4^, Veronica Macchi^1,3^, Monica Dettin^3,6^, Raffaele De Caro^1,3,^*, and Andrea Porzionato^1,3^

^1^Department of Neurosciences, Section of Human Anatomy, University of Padova, Via Aristide Gabelli 65 - 35127 Padova, Italy

^2^Department of Cardiac, Thoracic and Vascular Science and Public Health, University of Padova, Via Nicolò Giustiniani 2 - 35128 Padova, Italy

^3^L.i.f.e.L.a.b. Program, Consorzio per la Ricerca Sanitaria (CORIS), Veneto Region, Via Nicolò Giustiniani 2 - 35128 Padova, Italy

^4^Plastic and Reconstructive Surgery Unit, University of Padova, Via Nicolò Giustiniani 2 - 35128 Padova, Italy

^5^Department of Civil, Environmental and Architectural Engineering University of Padova, Via Francesco Marzolo 9 - 35131 Padova, Italy

^6^Department of Industrial Engineering University of Padova, Via Gradenigo 6/a - 35131 Padova, Italy

*Corresponding Author.

E-mail address: [raffaele.decaro@unipd.it](mailto:raffaele.decaro@unipd.it)

**Supplementary Information**


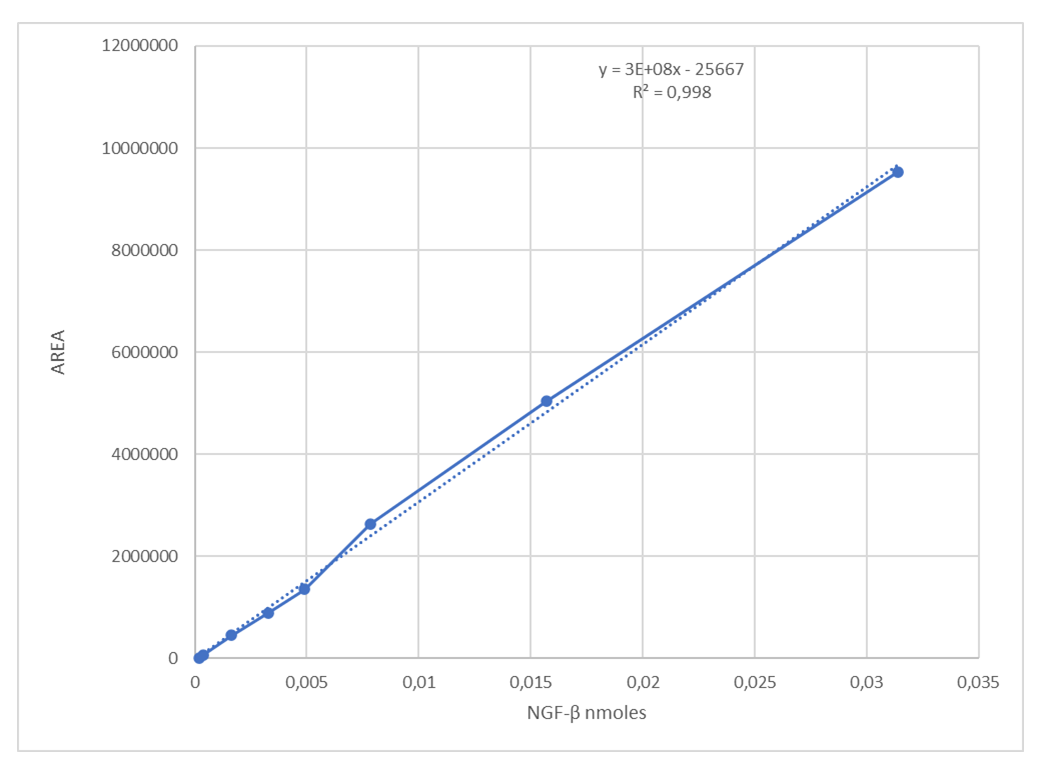


**S1.** Calibration curve (peak area versus nmoles of Nerve Growth Factor - NGF)


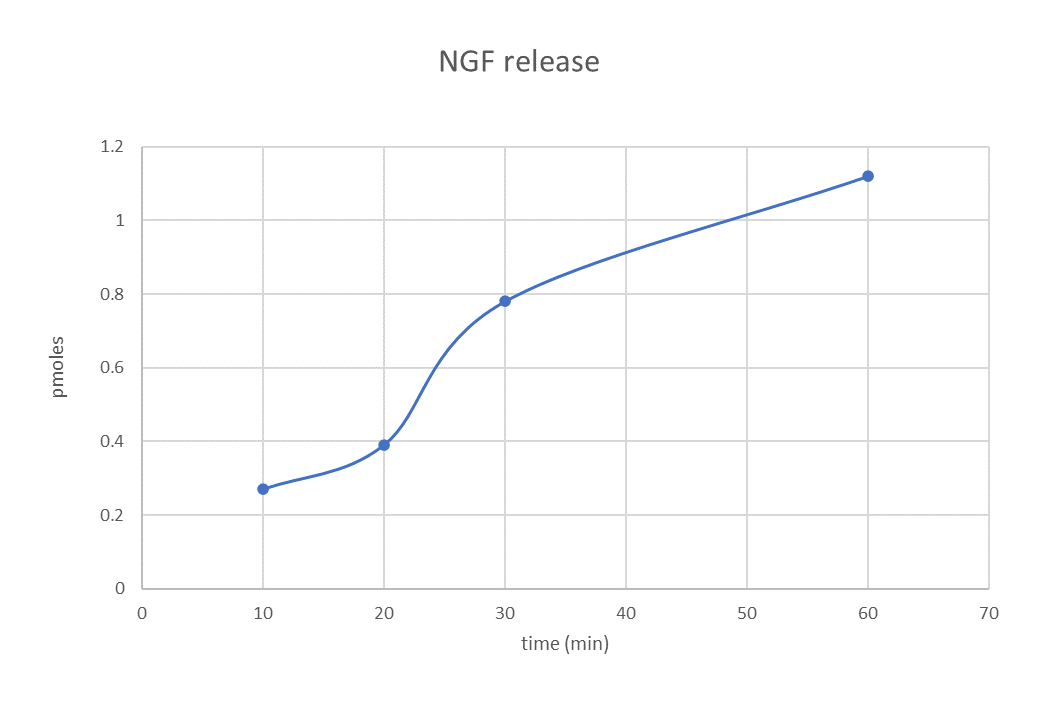


**S2.** Release profile of Nerve Growth Factor - NGF over 24 h
